# A next generation of the schema therapy model of personality pathology: A cross-cultural and international study protocol

**DOI:** 10.1371/journal.pone.0332723

**Published:** 2026-06-12

**Authors:** Freideriki Carmen Mamali, Marleen Rijkeboer, Dylan Molenaar, Sophie A. Rameckers, Marta Panzeri, Arnoud Arntz, Christopher W. Lee, Robert Brockman, Daniela Ho Tan, Muhammad Kamruzzaman Mozumder, Tarun Kanti Gayen, Dmitry Dyakov, Yuliya Stabrouskaya, Amirov Gatam, Vladislav Lobanovich, Sofiya Kanavina, Veronika Romanovich, Olkhouka Artsiom Andreevich, Natallia Tkhoryk, Aliaksandra Shabaikovich, Eva Dierckx, Els Pauwels, Kristina Eggermont, Fabiola Rodrigues Matos, Renan Puléo Barela, Lilian Queiroz Frossard, Irina S. Lazarova, Krista Peneva, Atanaska Katrandjieva, Anna St. Lazarova, Yawen Zhu, Baozhen Lyu, Yuhan Liu, Libo Li, Hongxia Qin, Xiaowei Guo, Xin Hu, Songhao Zhang, Olivia Y. T. Ma, Aled L. Y. Tang, Stine Bjerrum Moeller, Ida-Marie T. P. Arendt, Bo Bach, Jacob Meline, Géraldine Tapia, Katia M’bailara, Ketevan Abdushelishvili, Archil Begiashvili, Natia Badrishvili, Ana Gvinianidze, Lana Akopashvili, Irma Khabazi, Johannes Kopf-Beck, Cosima Leithne, Fritz Renner, Samy Egli, Sarah M. Quaatz, Ioannis A. Malogiannis, Eleni Giannoulis, Gregoris Simos, Zsolt Unoka, Lajos Simon, Viktoria Simon, Manjula Munivenkatappa, Paulomi Matam Sudhir, Tavleen Kaur Kohli, Apoorva Shrivastava, Arpita Alok Misra, Anisha Khanna, John Philip Louis, Naomi Maurilla Santoso, Theresia Indira Shanti, Laurentius Sandi Witarso, Maryam Hedayati, Adeleh Samimi, Saade Malekasgar, Barbara Basile, Ilona Krone, Agnese Orupe, Julija Gecaite-Stonciene, Justina Pociunaite, Huey Jing Renee Tan, Edward Chan, Veerathayalani Heimkumar, Nadia Kadri, Saadia Karroumi, Fatima Zahra Chentaf, Naima Ezzahiri, Hind Rachidi, Soukaina Bouchebti, Hind Bouidar, Wydad Hikmat, Fouzia Chroukate, Zainaib Ennaciri, Jens C. Thimm, Veronica Lorentzen, Jarosław M. Michałowski, Agnieszka E. Popiel, Dorota Mącik, Weronika Maria Browarczyk, Maria do Céu Salvador, Ancuța Elena Păduraru, Suzana E. Semeniuc, Paula I. Stroian, Irina Șubredu, Alexandra Yaltonskaya, Darya Maryasova, Olga Titova, Natalia Gegel, Natalia Gorodentseva, Maria Skryabina, Alena Plichko, Olga Pavlova, Svetlana Prokaeva, Viktoryia Malashchanka, Hui Ling Michelle Neo, Dan J. Stein, Waseem Hawa, Jean-Pierre Hartmann, Eunhee Lee, Younghee Song, Rocío Gómez-Juanes, Esther Calvete, Jordi Cid, Ralph E. Schmidt, Stephanie Homan, Chaiyun Sakulsriprasert, Darunee Phukao, Ratipan Thawornwutichat, Welika Mamoon, Anuk Chandraungs, Nanthaka Supreeyaporn, Nathan Bachrach, Marije Keulen-de Vos, Jill Lobbestael, Eline Dorrestijn, Julie Krans, Duygu Yakin, Deniz Baştak, Aliye Canan Taşlioğlu Sayiner, Başak İnce, Stephanie Tasilaridis, Mine Ergelen, Fiona C. Kennedy, Christopher D. J. Taylor, Michael B. First, George Lockwood, Zachary Appenzeller, Alice R. Norton, Elizabeth Seeley-Wait, Maree J. Abbott, Karina L. Allen, Surilena Hasan, Sergio Alejandro Morales Hernández, Abdelrahman Mohamed Sharaf, Florian Alexander Ruths

**Affiliations:** 1 Department of Psychology, Faculty of Social and Behavioural Sciences, University of Amsterdam, Amsterdam, the Netherlands; 2 Department of Clinical Psychological Science, Faculty of Psychology and Neuroscience, Maastricht University, Maastricht, the Netherlands; 3 Department of Developmental Psychology and Socialisation, Padua University, Padova, Italy; 4 Faculty of Health and Medical Sciences, University of Western Australia, Crawley, Western Australia, Australia; 5 Graduate School of Health, University of Technology Sydney, Sydney, New South Wales, Australia; 6 Faculty of Science, The University of Sydney, Sydney, Australia; 7 Department of Clinical Psychology, University of Dhaka, Dhaka, Bangladesh; 8 Institute of Psychology, Belarusian State Pedagogical University named after Maxim Tank, Minsk, Belarus; 9 Minsk CBT Center, Minsk, Belarus; 10 Regional State Budgetary Healthcare Institution “Irkutsk City Hospital #6”, Irkutsk, Russia; 11 Veronika Romanovich, Private Practice, Graz, Austria; 12 Healthcare Institution “Brest City Policlinic №2”, Brest, Belarus; 13 Oncological dispensary of Baranovichi Healthcare institution «Baranovichi central polyclinic», Baranovichi, Belarus; 14 Institute of Advanced Training and Retraining, Baranovichi State University, Baranovichi, Belarus; 15 Aliaksandra Shabaikovich, Private Practice, Minsk, Belarus; 16 Department of Psychology, Vrije Universiteit Brussel, Brussels, Belgium; 17 Psychiatric Clinic, Alexianen Zorggroep Tienen, Tienen, Belgium; 18 Psychologenpraktijk De Vest, Tienen, Belgium; 19 Faculty of Psychology and Educational Sciences, KU Leuven, Leuven, Belgium; 20 Child and Youth Institute, KU Leuven, Leuven, Belgium; 21 Department of Health and Psychology, Minas Gerais State University, Ituiutaba, Minas Gerais, Brazil; 22 Mindfluence Clinic, São Paulo, São Paulo, Brazil; 23 Lilian Frossard Institute, Blumenau, Santa Catarina, Brazil; 24 Psychosocial Rehabilitation Department, State Psychiatric Hospital “Sv. Iv. Rilski”, Novi Iskar, Sofia, Bulgaria; 25 Outpatient Mental Health Clinic “Adaptacia”, Sofia, Bulgaria; 26 Private Practice, Sofia, Bulgaria; 27 Sofia University “St. Kliment Ohridski”, Department of Classical Philology, Sofia, Bulgaria; 28 Key Laboratory of Behavioral and Mental Health of Gansu Province, School of Psychology, Northwest Normal University, Lanzhou, Gansu, China; 29 College of Science and Technology, Ningbo University, Ningbo, Zhejiang, China; 30 Department of Mental Health, College of Humanities, Anhui Science and Technology University, Chuzhou, Anhui, China; 31 School of Management, Shanghai University of International Business and Economics, Shanghai, China; 32 Private Practice, Fuzhou, Fujian, China; 33 Private Practice, Beijing, China; 34 Clinical Psychological Services, New Life Psychiatric Rehabilitation Association, Hong Kong SAR, China; 35 Danish National Center of Psychotraumatology, Department of Psychology, University of Southern Denmark, Odense, Denmark; 36 Mental Health Services in the Region of Southern Denmark, Department of Trauma and Torture Survivors, Vejle, Denmark; 37 Department of Psychology, Faculty of Social Sciences, University of Copenhagen, Copenhagen, Denmark; 38 Slagelse Psychiatric Hospital, Region Zealand, Slagelse, Denmark; 39 Child and Adolescent Psychiatric Clinic, Region Zealand, Holbæk, Denmark; 40 Laboratory of Psychology, UR4139, University of Bordeaux, Bordeaux, France; 41 Caucasus School of Humanities and Social Sciences, Caucasus University, Tbilisi, Georgia; 42 Faculty of Psychology and Educational Science, Department of Psychology, Tbilisi State University, Tbilisi, Georgia; 43 Tbilisi Family Mental Health Center, Tbilisi, Georgia; 44 Department of Psychiatry and Narcology, Tbilisi State Medical University, Tbilisi, Georgia; 45 Department of Social and Human Sciences, Faculty of Psychology, Gr. Robakidze University, Tbilisi, Georgia; 46 Natia Badrishvili, Private Practice, Tbilisi, Georgia; 47 Psychotherapy House “Kamara”, Tbilisi, Georgia; 48 Faculty of Humanistic and Social Sciences, Gr. Robakidze University, Tbilisi, Georgia; 49 Faculty of Medicine, European University, Tbilisi, Georgia; 50 Faculty of Medicine, UNIK, Kutaisi University, Kutaisi, Georgia; 51 Caucasus Medical Center, Tbilisi, Georgia; 52 M. Iashvili Children’s Central Hospital, Tbilisi, Georgia; 53 Neurodevelopment Center, Tbilisi, Georgia; 54 Civil Society Foundation, Tbilisi, Georgia; 55 Department of Psychology, LMU Munich, Munich, Germany; 56 Clinical Psychology and Psychotherapy Unit, Freiburg University, Freiburg, Germany; 57 Max Planck Institute of Psychiatry, Research Clinic, Department Clinical Translation, Munich, Germany; 58 Institute of Psychology, University of the Bundeswehr, Munich, Germany; 59 Specific Sector of Personality Disorders, First Department of Psychiatry, Eginition Hospital, Medical School, National and Kapodistrian University of Athens, Athens, Greece; 60 Neuropsychological and Psychometric Laboratory, Specific Sector of Personality Disorders, First Department of Psychiatry, Eginition Hospital, Medical School, National and Kapodistrian University of Athens, Athens, Greece; 61 Department of Education and Social Policy, University of Macedonia, Thessaloniki, Greece; 62 Department of Psychiatry and Psychotherapy, Semmelweis University, Budapest, Hungary; 63 Department of Clinical Psychology, National Institute of Mental Health and Neurosciences, Bangalore, India; 64 Center for Mental Health, Pune, India; 65 Manoshanti Clinic, Pune, India; 66 The Healing Space and TherapHeal, Kolkata, India; 67 Pusat Keluarga Dan Kaunseling Sdn. Bhd., Pulau Pinang, Malaysia; 68 Faculty of Medicine, National University of Malaysia, Kuala Lumpur, Malaysia; 69 Louis Family Services LLC, Lubbock, Texas, United States of America; 70 Faculty of Psychology, Atma Jaya Catholic University of Indonesia, Jakarta, Indonesia; 71 Segal Psychological Services and Counseling Center, Tehran, Iran; 72 Cognitive-Behavioral Department, Siavoushan Psychological Center, Tehran, Iran; 73 University of Science and Culture, Tehran, Iran; 74 School of Cognitive Psychotherapy, Italian Academy of Schema Therapy, Rome, Italy; 75 Department of Health Psychology and Education, Riga Stradins University, Riga, Latvia; 76 Faculty of Education Sciences and Psychology, University of Latvia, Riga, Latvia; 77 Laboratory of Behavioral Medicine, Neuroscience Institute, Lithuanian University of Health Sciences, Kaunas, Lithuania; 78 Department of Health Psychology, Faculty of Public Health, Lithuanian University of Health Sciences, Kaunas, Lithuania; 79 Department of Psychiatry and Mental Health, Hospital Kajang, Kajang, Selangor, Malaysia; 80 Amarantine Clinic, Wilayah Persekutuan, Kuala Lumpur, Malaysia; 81 Psychotherapy Department, International Psychology Centre, Kuala Lumpur, Malaysia; 82 Psychotherapy Departments, International Psychology Centre and Complementary Medicine University, Kuala Lumpur, Malaysia; 83 Moroccan Institute of Cognitive Behavioural Therapy, Casablanca, Morocco; 84 University Psychiatric Center, Marrakech, Morroco; 85 Department of Psychology, UiT The Arctic University of Norway, Tromsø, Norway; 86 Poznan Laboratory of Affective Neuroscience, Department of Psychology and Law, SWPS University, Poznań, Poland; 87 Institute of Psychology, SWPS University, Warsaw, Poland; 88 Institute of Psychology, John Paul II Catholic University of Lublin, Lublin, Poland; 89 University of Coimbra, Center for Research in Neuropsychology and Cognitive and Behavioral Intervention (CINEICC), Coimbra, Portugal; 90 Department of Psychology, Faculty of Psychology and Education Sciences, “Alexandru Ioan Cuza” University of Iași, Iași, Romania; 91 Socola Institute of Psychiatry, Iași, Romania; 92 International Institute for the Advanced Study of Psychotherapy and Applied Mental Health, Babes-Bolyai University, Cluj-Napoca, Romania; 93 Becoming Center, Iași, Romania; 94 Moscow Institute of Schema Therapy, Moscow, Russia; 95 A.Plichko, Private Practice, Moscow, Russia; 96 O. Pavlova, Private Practice, Saint-Petersburg, Russia; 97 S. Prokaeva, Private Practice, Moscow, Russia; 98 V. Malashchanka, Private Practice, Poznań, Poland; 99 National University Hospital, Kent Ridge, Singapore; 100 Department of Psychiatry and Neuroscience Institute, SAMRC Unit on Risk and Resilience in Mental Disorders, University of Cape Town, Cape Town, South Africa; 101 Department of Psychiatry and Mental Health, Division of Psychotherapy, University of Cape Town, Cape Town, South Africa; 102 Western Cape, Department of Health and Wellness, Cape Town, South Africa; 103 Department of Psychology, Kyungnam University, Changwon, Gyeongsangnam, South Korea; 104 Y Psychological Counseling Center Co, Changwon, Gyeongsangnam, South Korea; 105 Department of Psychiatry, Son Espases University Hospital, Palma, Spain; 106 Department of Medicine, University of the Balearic Islands, Palma, Spain; 107 Faculty of Health Sciences, University of Deusto, Bilbao, Spain; 108 Institut d’Investigació Biomèdica de Girona Dr. Josep Trueta (IDIBGI) – IAS, Salt, Girona, Spain; 109 Department of Adult Psychiatry and Psychotherapy, Psychiatric University Clinic Zurich and University of Zurich, Zurich, Switzerland; 110 Department of Psychology, University of Geneva, Geneva, Switzerland; 111 Department of Psychology, Experimental Psychopathology and Psychotherapy, University of Zurich, Zurich, Switzerland; 112 Department of Psychology, Faculty of Humanities, Chiang Mai University, Chiang Mai, Thailand; 113 Department of Society and Health, Faculty of Social Sciences and Humanities, Mahidol University, Nakhon Pathom, Thailand; 114 Faculty of Liberal Arts, Maejo University, Chiang Mai, Thailand; 115 Ooca Clinic, Bangkok, Thailand; 116 Department of Educational Foundations and Development, Faculty of Education, Chiang Mai University, Chiang Mai, Thailand; 117 Department of Trauma Related and Personality Disorders, GGZ Oost Brabant, Helmond, the Netherlands; 118 Department of Medical and Clinical Psychology, Tilburg University, Tilburg, the Netherlands; 119 De Rooyse Wissel, Venray, the Netherlands; 120 Department of Clinical Psychological Science, Maastricht University, Maastricht, the Netherlands; 121 Vitaal GGZ, Nijkerk, the Netherlands; 122 Behavioural Science Institute, Radboud University, Nijmegen, the Netherlands; 123 Pro Persona Research, Nijmegen, the Netherlands; 124 Faculty of Psychology and Educational Sciences, Department of Clinical Psychology, KU Leuven, Leuven, Belgium; 125 Department of Human and Social Sciences, University of Bergamo, Bergamo, Italy; 126 Department of Psychology, Faculty of Economics, Administrative and Social Sciences, Bahçeşehir University, Istanbul, Türkiye; 127 Centre for Research in Eating and Weight Disorders, Department of Psychological Medicine, Institute of Psychiatry, Psychology, and Neuroscience, King’s College London, London, United Kingdom; 128 Department of Psychology, Faculty of Economics Administrative and Social Sciences, Işik University, Istanbul, Türkiye; 129 Erenköy Mental Health and Nervous Diseases Training and Research Hospital, University of Health Sciences, Istanbul, Türkiye; 130 School of Psychology, Southampton University, Southampton, United Kingdom; 131 GreenWood Mentors Ltd, Ryde, United Kingdom; 132 Department of Psychology, Faculty of Science, The University of Sheffield, Sheffield, United Kingdom; 133 Department of Psychiatry, Columbia University Vagelos College of Physicians and Surgeons, New York, New York, United States of America; 134 Biometrics Department, New York State Psychiatric Institute, New York, New York, United States of America; 135 Schema Therapy Institute Midwest, Kalamazoo, Michigan, United States of America; 136 Louis A. Faillace, MD, Department of Psychiatry and Behavioral Sciences, McGovern Medical School, The University of Texas Health Science Center at Houston (UTHealth Houston), Houston, Texas, United States of America; 137 School of Psychology, Clinical Psychology Unit, Faculty of Science, The University of Sydney, Sydney, Australia; 138 Centre for Research in Eating and Weight Disorders, Institute of Psychiatry, Psychology and Neuroscience, King’s College London, London, United Kingdom; 139 Eating Disorders Outpatients Service, South London and Maudsley NHS Foundation Trust, London, United Kingdom; 140 Faculty of Medicine, Atma Jaya Catholic University of Indonesia, Jakarta, Indonesia; 141 Instituto Mexicano de Terapia de Esquemas, Mexico City, Mexico; 142 Safe Haven Clinic, Alexandria, Egypt; 143 Maudsley Hospital, South London and Maudsley NHS Foundation Trust, London, United Kingdom; University of Kansas School of Medicine Wichita, UNITED STATES OF AMERICA

## Abstract

**Background:**

Personality disorders are highly prevalent worldwide imposing substantial personal and social challenges. Schema Therapy is an effective psychotherapeutic approach for personality pathology and other complex characterological problems. New scientific insights prompted a re-evaluation of its theoretical underpinnings leading to a reformulated Schema Therapy theory. Furthermore, the assumed cross-cultural universality of the Schema Therapy model has not been tested.

**Aims:**

This project has two primary aims: (1) To develop revised instruments based on the reformulated theory that are psychometrically sound and valid across diverse cultures and languages. (2) To validate the Schema Therapy-related constructs and their inter-relationships across cultures.

**Methods:**

New draft versions of the Young Schema Questionnaire, Schema Coping Inventory, and Schema Mode Inventory were developed. Before dissemination, these instruments will undergo rigorous psychometric evaluation to refine item sets and ensure linguistic and conceptual consistency. A minimum of 100 adult mental health patients and 100 non-patients from each participating country will complete the revised instruments. Sociodemographic and mental health-related variables will also be assessed. Statistical analyses will evaluate (a) internal consistency, (b) unidimensionality, (c) cross-cultural invariance, (d) factorial validity (if possible), and (e) known-group validity. Malfunctioning items will be deleted, and subscales will be shortened, if possible, targeting internal consistency of ≥.80.

**Expected outcomes:**

This study is expected to yield optimized versions of the three instruments aligned with the reformulated theory. These findings will inform subsequent international studies to further assess the structural and cross-cultural validity of the revised scales. The resulting empirically validated scales will be openly accessible, facilitating worldwide utilization.

**Discussion:**

This protocol outlines the first international study based on the reformulated theory, aiming to extend the psychopathological coverage and enhance the cross-cultural application of evidence-based treatments for personality pathology. Results will be disseminated through peer-reviewed publications and conference presentations. Potential limitations are discussed.

## Introduction

Personality disorders (PDs) have attained international recognition as a significant mental health concern due to being associated with many adverse outcomes including premature mortality, an incremented risk of various physical and mental health comorbidities, as well as substantial individual and societal costs [1–4]. Several community-based epidemiological studies indicate that the prevalence of any PD ranges from 4.4% to 21.5% across Western nations [4,5], while PDs appear to affect around 6% to 7.8% of the global population [6,7]. Notably, lower prevalence rates have been observed within certain Asian countries. For instance, in the general population of India, the prevalence of PDs was previously estimated at 1.07% [7–9]. Yet, limitations in survey methodologies, including the use of less sensitive screening tools, reliance on single informants, and limited attention to comorbidity or dual diagnoses, likely contribute to the underreporting of PD prevalence in these contexts. Cultural influences and societal stigma may further inhibit disclosure or lead to the under-recognition of certain traits as clinically significant. Additionally, nosological differences across diagnostic systems can further obscure cross-cultural comparisons. For example, avoidant and borderline PDs are not included in the Chinese Classification of Mental Disorders, potentially limiting their identification in epidemiological research [8,10–12]. Taken together, these inconsistencies make it difficult to draw meaningful comparisons across countries and suggest that the observed low prevalence rates in some regions may reflect methodological underestimation rather than true epidemiological differences. In clinical settings worldwide, the prevalence estimates stand significantly high; amongst those diagnosed with a psychiatric disorder approximately one-third meet the diagnostic criteria for (at least) one PD [13,14].

Introduced in the 1990’s by Jeffrey Young [15], Schema Therapy (ST) has evolved into a highly effective and cost-effective psychotherapeutic approach tailored to address pervasive and persistent mental health problems including, but not limited to, personality and chronic anxiety disorders, as well as persistent depression [16–21]. Originally developed to address limitations of traditional cognitive therapy for treating chronic characterological issues, ST emphasizes the crucial role of unmet core emotional needs in forming enduring maladaptive schemas which underlie persistent psychological problems and maladaptive coping [15]. While initially focused on modifying these schemas, ST later expanded to include the schema mode model, aimed at addressing the shifting emotional states and coping patterns seen in complex disorders such as borderline PD [22]. Additional key milestones in the evolution of the ST framework include the development of specific schema modes for borderline, cluster C and several other PDs, as well as forensic psychopathology [23–27].

The interest in ST has grown worldwide and ST is nowadays applied in many countries. The widespread use of ST notwithstanding, there remain notable gaps in the theoretical foundations of the model and its implementation [28,29]. Specifically, while the schema mode model has undergone several extensions, a well-defined theoretical framework to systematically underpin these expansions is largely missing [28]. In addition, the current ST framework is somewhat limited in scope, primarily focusing on an incomplete range of personality pathology, while the cross-cultural assessment of the core ST-related constructs has yet to receive robust empirical support. To illustrate, while substantial evidence supports the trans-cultural applicability of early maladaptive schemas—demonstrated by their presence across diverse populations and cultural contexts [e.g., 30–34]—cross‑cultural differences in schema endorsement and in coping with schema activation have also been documented [e.g., 30,35,36]. Conversely, research exploring how schema modes apply to non-Western populations is rather scarce. What available evidence there is provides some preliminary support for the construct’s cross-cultural validity in certain Eastern and Asian contexts [e.g., 37–40], though more comprehensive and large-scale investigations are required to confirm these findings. Lastly, although ST is a Western-developed approach, emerging findings suggest that the framework holds promise across cultures, while also highlighting that cultural factors may influence client engagement and clinical application [37,41–43]. These findings underscore the need to examine whether ST‑related constructs are consistent across cultural contexts. Such efforts depend on the availability of assessment instruments that are carefully adapted—both linguistically and conceptually—so that items convey comparable meanings across languages and cultures.

Cultural factors exert a substantial influence in moulding the clinical expression, conceptualization, and treatment of mental health disorders and efforts to integrate culturally responsive, evidence-based frameworks within mental health services have been increasing [44,45]. The scope of this study is to construct and validate revised ST assessment instruments, ensuring their applicability across diverse cultural contexts. Developing culturally sensitive and linguistically appropriate assessment instruments is essential for evaluating the cultural suitability of the ST model. This further facilitates the advancement of trans-cultural research focused on developing evidence-based cultural adaptations of schema-focused interventions, particularly in regions where such research is currently limited.

### The psychopathology model underlying schema therapy

The theoretical framework of the ST model posits that individuals inherently possess a set of five core emotional needs: secure attachments to others; autonomy, competence, and sense of identity; freedom to express needs and emotions; spontaneity and play; and realistic limits and self-control. These needs are believed to be universally present, albeit to varying degrees, in all humans. The frustration or inadequate fulfilment of these fundamental psychological needs during early developmental stages (i.e., childhood or adolescence) can lead to dysfunctional mental representations concerning oneself and relationships with others, termed ‘Early Maladaptive Schemas’ (EMSs). These schemas are viewed as enduring trait-like patterns that are elaborated throughout a person’s lifetime rendering the individual psychologically vulnerable to the development of longstanding characterological problems (e.g., PDs) and other forms of psychopathology [22]. To illustrate, if an individual has recurrently encountered severe criticism and rejection during their early life, an EMS of ‘Defectiveness/Shame’ is likely to develop. This schema will significantly influence their self-perception and their perception of others, causing them to feel inherently flawed, inferior, and/or worthless to such an extent that they believe no one could love them if their imperfections were revealed.

Despite their inflexible and pervasive nature, EMSs are not constantly active but rather triggered by the dynamic interplay of life events, which may vary from moment to moment. The suspected or actual activation of an EMS signifies the thwarting of a core emotional need, resulting in intense distressing emotions such as guilt, anger, shame, and helplessness. According to Young [15,22], individuals with mental health problems respond to the (threat of) activation of EMSs by consciously or (often) unconsciously employing (one or a combination of) three maladaptive ways of coping, namely surrender, avoidance, and overcompensation. Surrender, referred to as ‘resignation’ by Arntz et al. [28], entails the individual resigning themselves to the EMS by endorsing the cognitive content of the schema as being true and exhibiting feelings and behaviours that entirely comply with the EMS. Avoidance pertains to efforts aimed at escaping or blocking the EMS activation by evading the mental content or emotions linked to the schema, and by engaging in behaviours intended to shield oneself from complete EMS activation. Lastly, overcompensation, termed as ‘inversion’ by Arntz and colleagues [28], involves the individual believing that the opposite of the EMS is true and is characterized by thoughts, emotions, and behaviours that serve to prove the contrary of the schema.

The final pivotal construct in the ST framework is that of ‘Schema Modes’ (SMs), which was introduced by Young and colleagues [22] to conceptualize the sudden shifts in emotional-cognitive features frequently observed in individuals with severe personality pathology. According to the definition, an SM is comprised of a combination of active EMSs and ways of coping that reflect an individual’s predominant momentary cognitive, affective, and behavioural state [22,46]. The original mode model defined 10 primary modes, classified into four principal categories: (a) dysfunctional child modes (resulting from a surrender coping response to EMS activation); (b) maladaptive coping modes (corresponding to avoidant or overcompensating coping with schema activation); (c) dysfunctional parent modes (reflecting the internalization of unhealthy parental behaviours towards the child); and (d) healthy modes (representing adaptive functioning). Over the past decade, the SM model has been extended to also encompass additional distinctive modes and mode sets that are central in specific PDs or other diagnostic entities [25,28,46].

Thus, in summary, ST theory postulates that modes result from the way people cope with the activation of specific EMSs. In other words, the way of coping with the activation of a specific EMS leads to a specific SM. If avoidance or inversion (formerly: overcompensation) coping with EMS activation dominates, a coping mode results: the feelings, thinking, and behaviour of the individual are dominated by the way of coping. If there is resignation (formerly: surrender) coping to EMS activation, child, or internalized parent modes result.

The three basic elements of the ST theory outlined above are often assessed with self-report instruments. Specifically, the presence and intensity of EMSs are typically assessed through the Young Schema Questionnaire (YSQ) [47]. The YSQ has undergone multiple iterations, resulting in several versions of the instrument, and has been extensively employed in both research studies and as part of the therapeutic assessment conducted in clinical settings. Further, the Schema Mode Inventory (SMI-1) [27,48] and the SMI-2 [25] were devised to assess various SMs. Lastly, the Schema Coping Inventory (SCI) [49] was developed to assess the three unhealthy ways of coping with EMS activation as defined by Young and colleagues [22]. It should be noted that the SCI was designed to facilitate research endeavours aimed at empirically examining the postulated relationships between schema coping strategies and other ST-related constructs, rather than its application in clinical practice.

### Recent developments in the schema therapy model

Despite the growing interest and worldwide utilization of the ST model, its theoretical underpinnings are devoid of empirical validation of several critical assumptions [28,29]. Recently, Arntz and colleagues [28] proposed a reformulated theory, aimed at rectifying several limitations within the theoretical basis of the ST model, while also expanding its applicability across diverse cultural contexts and clinical disorders.

According to the authors, Young’s initial theorization on the development of EMSs was predicated on the hypothesis that there exist five universal emotional needs, the fulfilment of which is crucial for optimal psychological well-being. As underlined by Young, the five hypothesized needs were formulated based on his reading of pertinent theories and clinical observations, in the absence of a comprehensive theoretical framework of core developmental needs [22]. Dweck’s recent theory on motivation, personality, and development [50] indicates that certain fundamental emotional needs might have been overlooked in the ST framework. Drawing upon Dweck’s theory and considering clinical observations and empirical evidence, Arntz and colleagues proposed the integration of the need for self-coherence (and the respective schemas of ‘Incoherent Identity’ and ‘Incomprehensible World’) in the taxonomy of core psychological needs and the list of EMSs initially outlined by Young. The need for self-coherence refers to the fundamental desire to experience oneself as psychologically whole, as opposed to fragmented or diffused. According to Dweck, it serves as a central organizing need, integrating experiences and goals to maintain a sense of identity and personal meaning [50]. An unmet need for self-coherence may manifest as a pervasive sense of disconnection and alienation from oneself and others, existential confusion, and a destabilized sense of identity which may culminate in dissociative experiences or, in severe instances, psychotic states [28]. Such disruptions in the sense of self have been both phenomenologically and empirically linked to borderline PD as well as a broad spectrum of other PD–related symptomatology, to which ST is widely applied [e.g., 51–53]. Accordingly, incorporating this need and its corresponding schemas into the ST framework would allow for a more nuanced conceptualization and treatment of self-related disturbances across personality and other complex disorders.

Moreover, the international workgroup considered that the need for fairness (and its corresponding schema of ‘Unfairness’) was missing in ST theory, hence these were also proposed to be added (for a detailed description see [28]). The sense of fairness emerges as a fundamental human concern, observable from infancy and early childhood [54,55]. Humans share this sensitivity with animals that collaborate on an individual basis, and ethologists have argued that the need for fairness—and the primary emotional response to unfairness, anger— serves the survival changes of such species [56]. Perceived unfairness has been consistently associated with adverse physical and mental health outcomes, including psychosomatic complaints, anxiety and depressive symptoms, as well as maladaptive behaviours such as aggression and vengefulness [57–64]. Further, injustice sensitivity is thought to exacerbate interpersonal dysfunction in borderline PD [65] and is linked to narcissistic traits [66,67]. Lastly, recent findings indicate that Imagery Rescripting—an intervention widely integrated into ST—demonstrates promise in alleviating emotional distress associated with perceived unfairness [68]. Given its clinical relevance, the inclusion of the construct of ‘Unfairness’ within the current ST framework is considered warranted.

Furthermore, considering the proposed revisions to the YSQ put forth by Yalcin and colleagues [69], the construct of ‘Emotional Inhibition’ was bifurcated into two distinct subscales: ‘Emotional Constriction’, denoting an excessive control over and disconnection from emotions, and ‘Fear of Losing Control’, representing the belief that failure to control emotions will lead to severe repercussions. Similarly, the ‘Punitiveness’ subscale was subdivided into ‘Internal Punitiveness’ and ‘External Punitiveness’. The former encompasses self-directed punitiveness, while the latter embodies the belief that others should be severely penalized for their mistakes and imperfections. These revised constructs were derived from rigorous psychometric analyses and align with earlier findings [70,71]. The proposed subscales have demonstrated good to excellent internal consistency and predictive validity [69,72]. Collectively, this evidence supports the multidimensional nature of these original YSQ subscales, suggesting that previous versions of the instrument may have been limited in capturing the full complexity and nuance of the measured constructs. Clinically, this scale division also highlights meaningful distinctions: ‘Fear of Losing Control’ is linked to emotional instability and heightened threat sensitivity, commonly observed in borderline PD [73], post-traumatic stress disorder [74], and anxiety disorders [75]. In contrast, ‘Emotional Constriction’ reflects an overcontrolled style seen in major depressive disorder and cluster C PDs [34,76,77]. Similarly, the distinction between ‘Internal’ and ‘External Punitiveness’ corresponds to internalising versus externalising psychopathological processes, characterizing different clinical presentations [69]. Taken together, these refinements enhance both the psychometric precision and clinical relevance of the YSQ, addressing key limitations in earlier versions of the instrument.

In addition, Arntz et al. [28] refined the definition of the ways of coping with EMS activation and introduced new SMs based on a systematic application of the reformulated theory. More specifically, as mentioned before, the coping strategies of ‘surrender’ and ‘overcompensation’ were renamed as ‘resignation’ and ‘inversion’ respectively. The objective of relabelling these concepts served three aims: (i) to underscore the intrapsychic function of coping with EMS activation, delineating it as an internal mental operation directed towards the EMS activation rather than a behavioural response aimed at an external threat; (ii) to mitigate the overlap between coping strategies and (internalizing vs externalizing) temperament dimensions; and (iii) to enhance the applicability of these maladaptive ways of coping across the entire range of EMSs. Moreover, the international workgroup concluded in hindsight that the theoretical rationale of the existing SCI fell short because a temperamental construct regarding how people deal with external frustration had been mixed with how people deal with the intrapsychic phenomenon of EMS activation. Hence, a new SCI had to be developed.

Finally, after systematically applying the reformulated ST model across all EMSs, a new taxonomy of SMs emerged (for a detailed description see [28]). A theoretical conceptualization of the proposed taxonomy of modes is provided in Table 1, where “Schema Mode” denotes those modes originating from the application of the reformulated ST model as outlined by Arntz et al. [28], whereas “Proposed Schema Mode” pertains to those modes suggested to result from this project.

**Table 1:** Theoretical conceptualization of the proposed taxonomy of schema modes.

EMSs and *need domain* (in italics)	Schema Mode	Proposed Schema Mode
	*Vulnerable Child Modes*	*Vulnerable Child Modes* ^a^
**EMSs in the *need domain Safety & Nurturance***		
Abandonment/Instability.	Abandoned Child	Disregarded Child
Mistrust/Abuse.	Abused Child	
Emotional Deprivation.	Lonely Child	
Defectiveness/Shame.	Inferior/Ashamed Child	
Social Isolation/Alienation.	Alienated/Misunderstood Child	
**EMSs in the *need domain Autonomy, Competence, & Identity***		
Dependence/Incompetence.	Dependent Child	Non-Autonomous Child
Vulnerability to Harm or Illness.	Anxious Child	
Enmeshment/Undeveloped Self.	Non-Individualized Child	
Failure.	Failed Child	
**EMSs in the *need domain Freedom to Express Needs, Opinions, & Emotions***		
Subjugation.	Intimidated Child	Subordinate Child
Self-Sacrifice.	Parentified Child	
Approval-/Recognition-Seeking.	Attention Demanding Child	
**EMSs in the *need domain Spontaneity & Play***		
Negativity/Pessimism.	Pessimistic Child	Constrained Child
Emotional Constriction.	Inhibited Child	
Fear of Losing Control.	Frightened/Panicking Child	
Internal Punitiveness.	Bad Child	
Unrelenting Standards/Hypercriticalness.	Over-Diligent Child	
Unrelenting Standards/Hypercriticalness.	Disappointing/Short-Falling Child	
**EMS in the *need domain Realistic Limits & Self-Control***		
Entitlement/Grandiosity.	No Vulnerable Child Modes, see below for associated Child Modes.	
Insufficient Self-Control/Self-Discipline.	No Vulnerable Child Modes, see below for associated Child Modes.	
**EMS in the *need domain Fairness***		
Unfairness.	Victimized Child	Victimized Child
**EMSs in the *need domain Self-Coherence***		
Incoherent Identity.	Confused Child	Confused Child
Incomprehensible World.	Disconnected Child	
	***Non-Angry Externalizing Child Modes***	***Non-Angry Externalizing Child Modes***
**EMSs**		
Insufficient Self-Control/Self-Discipline, Abandonment/Instability, Mistrust/Abuse, Emotional Deprivation, Defectiveness/Shame, Social Isolation/Alienation, Dependence/Incompetence, Vulnerability to Harm or Illness, Entitlement/Grandiosity, Approval-/ Recognition-Seeking, Internal Punitiveness, External Punitiveness, Unfairness.	Impulsive Child	Impulsive Child
Entitlement/Grandiosity.	Spoiled Child	Spoiled Child
Entitlement/Grandiosity.	Grandiose Child	Grandiose Child
Insufficient Self-Control/Self-Discipline.	Undisciplined Child	Undisciplined Child
	***Angry Child Modes***	***Angry Child Modes*** ^b^
**EMSs**		
All EMSs.	Angry Child	Angry Child
Self-Sacrifice.	Aggrieved Child	
Internal Punitiveness.	Protesting Child	
Entitlement/Grandiosity (or perhaps all EMSs, as with the Angry Child Mode).	Enraged Child	Enraged Child
Enmeshment/Undeveloped Self, Subjugation, Unrelenting Standards/ Hypercriticalness.	Rebellious Child	Rebellious Child
Enmeshment/Undeveloped Self, Insufficient Self-Control/Self-Discipline, Approval-/Recognition-Seeking, Unrelenting Standards/Hypercriticalness, Internal Punitiveness.	Sulking Child	Sulking Child
	***Norm-Setting Modes (Parental Modes)***	***Norm-Setting Modes (Parental Modes)***
**EMSs**		
External Punitiveness.	Punitive Lecturer	Punitive Lecturer
Internal Punitiveness.	Punitive Critic (Punitive Parent)	Punitive Critic (Punitive Parent)
Unrelenting Standards/ Hypercriticalness.	Demanding Critic (Demanding Parent)	Demanding Critic (Demanding Parent)
	***Avoidance Coping Modes***	***Avoidance Coping Modes***
**EMSs** ^c^		
	Detached Protector	Detached Protector
	Funny Protector	Funny Protector
	Angry Protector	Angry Protector
	Avoidant Protector	Avoidant Protector
	Compliant Surrender	Compliant Surrender
	Reassurance Seeker	Reassurance Seeker
	Detached Self-Soother	Detached Self-Soother
	Suspicious Over-Controller	Suspicious Over-Controller
	***Inversion Coping Modes***	***Inversion Coping Modes***
**EMSs**		
Abandonment/Instability, Subjugation, Dependence/Incompetence, Enmeshment/Undeveloped Self, Approval-/Recognition-Seeking, Self-Sacrifice.	Hyper-Autonomous	Hyper-Autonomous
Emotional Deprivation, Failure, Insufficient Self-Control/Self-Discipline.	Perfectionistic Over-Controller	Perfectionistic Over-Controller
Abandonment/Instability, Mistrust/Abuse, Subjugation, Unfairness.	Bully & Attack	Bully & Attack
Emotional Deprivation, Defectiveness/Shame, Social Isolation/Alienation, Emotional Constriction.	Attention- & Approval-Seeker	Attention- & Approval-Seeker
Defectiveness/Shame, Self-Sacrifice, Social Isolation/Alienation, Subjugation, Approval-/Recognition-Seeking.	Self-Aggrandizer	Self-Aggrandizer
Abandonment/Instability, Mistrust/Abuse, Emotional Deprivation, Failure, Defectiveness/Shame, Subjugation, Social Isolation/Alienation, Self-Sacrifice, Dependence/Incompetence, Vulnerability to Harm or Illness, Enmeshment/Undeveloped Self, Negativity/Pessimism, Fear of Losing Control.	Clown	Clown
Incoherent Identity, Incomprehensible World.	Pretender	Pretender
Abandonment/Instability, Unfairness.	Conning & Manipulation	Conning & Manipulation
Mistrust/Abuse, Unfairness.	Idealizer	Idealizer
Vulnerability to Harm or Illness.	Daredevil	Daredevil
Unrelenting Standards/Hypercriticalness.	Slacker/Oblomov	Slacker/Oblomov
Negativity/Pessimism.	Pollyanna/Over-Optimist	Pollyanna/Over-Optimist
External Punitiveness.	Over-Merciful	Over-Merciful
Internal Punitiveness.	Overly Self-Permissive	Overly Self-Permissive
Entitlement/Grandiosity.	Over-Humble	Over-Humble
Emotional Constriction.	Emotional Excessiveness	Emotional Excessiveness
Fear of Losing Control.	Emotional Daredevil	Emotional Daredevil
Unfairness.	Predator	Predator
Failure.	Winner	Winner
	***Functional/Adaptive Modes***	***Functional/Adaptive Modes***
**EMSs** ^d^		
	Happy Child	Happy Child
	Healthy Adult	Healthy Adult

^a^The Vulnerable Child modes are grouped into six categories —Disregarded Child, Non-autonomous Child, Subordinate Child, Constrained Child, Victimized Child and Confused Child— according to the core emotional need that relates to the EMSs. Please note that in the article by Arntz et al. [28], seven (instead of six) categories are erroneously mentioned.

^b^The Angry Child modes are grouped into four categories —Angry Child, Enraged Child, Rebellious Child, and Sulking Child—depending on the specific type of anger and the behavioural expressions associated with each.

^c^The Avoidance Coping modes are formulated independently of EMSs, considering that these modes primarily represent the way of coping with schema activation, rather than the underlying EMS. Hence, no specific EMSs are designated for these modes.

^d^No EMSs are listed since the Happy Child and Healthy Adult represent adaptive modes where an individual’s core emotional needs are currently met, or where a frustration of a need is dealt with in an adaptive way.

It is suggested that the reformulated model leads to wider applicability of the ST framework by offering an improved coverage of severe psychopathologies (e.g., schizoid personality disorder, dissociative identity disorder) and specific problems (e.g., oversensitivity to unfairness) that are currently not adequately accounted for in Young’s model.

### Study framework

In light of the recent advancements in the theoretical understanding of ST, a team of Dutch researchers and senior experts in ST, directed by Arntz and Rijkeboer, established an international consortium comprising researchers, therapists, and clinicians from various countries across the globe. The consortium represents a diverse assembly of experts actively engaged in the field of ST, hailing from more than 35 countries.

More specifically, eight workgroups were established to develop items for the revised questionnaires based on the reformulated theory of ST. In each workgroup, a designated chair undertook the responsibility of coordinating workgroup tasks (e.g., task division and allocation) and facilitating communication with the central investigators. The composition of the workgroups was purposefully heterogeneous, encompassing individuals (i.e., chairs and members) with varying backgrounds in language, culture, clinical and research expertise, as well as gender representation. This diversity allowed for consideration of how item content might be interpreted across different contexts during the drafting process. Six workgroups focused on developing items for subsets of schema modes, one workgroup on developing items for the new schemas (i.e., ‘Incomprehensible World’, ‘Incoherent Identity’, ‘Unfairness’), and one workgroup on developing items for a new SCI. Experts specialized in specific fields were assigned to workgroups tasked with drafting items relevant to their respective domains (e.g., forensic modes).

Further, to facilitate content overlap checks during the initial stages of the process, sets of to-be-developed subscales which posed a risk of potential overlap, were allocated to the same workgroup. In addition, workgroup members received specific guidelines, instructing them to consider both the construct to be assessed (e.g., EMSs not involving behaviours) and the rating scales (i.e., ensuring the items could be appropriately scored on the rating scale) while formulating the items. It should be noted that the rating scales utilized in the pre-existing questionnaires were modified. To illustrate, it was deemed that the anchor points of the 6-point Likert scale used in previous versions of the YSQ resulted in asymmetry, which was considered an undesirable feature. Specifically, the scale provided only two gradations of disagreement (‘*Completely untrue of me*’ and ‘*Mostly untrue of me*’), whereas four options reflected increasing levels of agreement, with the midpoint (‘*Slightly more true than untrue*’) oriented toward endorsement. This imbalance could introduce response bias and limit the scale’s capacity to accurately measure neutral or ambivalent responses. Similarly, the Likert scale utilized in previous versions of the SMI lacked a midpoint, offering six response options ranging from ‘*Never or almost never*’ to ‘*All of the time*’. While this forced-choice format may encourage decisiveness, it may also introduce certain psychometric limitations including forced-choice bias and increased measurement error which lead to a compromised test validity and reliability, particularly in culturally diverse samples with varying response styles [e.g., 78–80]. Although the literature on the use of a midpoint in questionnaire research is mixed [e.g., 80,81], our perspective is that, with adequate instruction and data analyses, a midpoint can significantly contribute to the test’s validity and the respondent’s involvement in the task (see the discussion in [82]). Therefore, taking into account psychometric as well as language and cultural considerations, it was decided to adopt new formats for the Likert scales. In particular, we implemented 7-point end-anchored scales, as attempts to formulate equivalent labels for each of the seven points in all the languages of the project were unsuccessful. This adjustment aimed to enhance the effectiveness of the response scales and address the aforementioned limitations.

The workgroups encompassed representatives from more than 25 languages, facilitating a comprehensive evaluation of item clarity and comprehensibility across various linguistic and cultural contexts in parallel. Representatives from each language were also tasked with conducting quality checks on translations of existing scales (i.e., SMI-1, SMI-2, and YSQ-3) in their respective languages. English was the “lingua franca” of the project. That is, workgroups communicated and discussed the initially developed items in English. Members began by drafting items in their native languages, which they then translated into English for discussion and refinement within their groups. Throughout the development process, members were also encouraged to identify and discuss any potential linguistic or cultural ambiguities. Subsequently, to ensure the accuracy of item wording—encompassing semantic, idiomatic, experiential, and conceptual equivalence—and to maintain cross-linguistic consistency, each workgroup member carried out translations from and back translations into English. Further, each workgroup included a native English speaker to ensure the precision and consistency of the English version of the items.

The newly formulated items were subjected to pilot testing to assess their comprehensibility in different languages, as well as their suitability for rating on the standard (i.e., 7-point) rating scale by interviewing a small sample of lay people. This first check was conducted by all workgroup members with all the items they developed in their workgroup. Concurrently, regular meetings with the chairs of all workgroups were held during the process of item development and pilot testing. These meetings brought to light several issues concerning the instructions used in pre-existing questionnaires. Consequently, to enhance comprehensibility and ensure a more accurate representation of the constructs under assessment, several revisions were implemented to the instructions of the YSQ, SCI, and SMI. Another aspect of revision pertained to the labelling of certain SMI and YSQ subscales. For instance, the ‘Merciful’ subscale introduced in Arntz et al.’s [28] position paper was renamed as ‘Over-Merciful’ to signify the dysfunctional aspect of the mode more accurately. Additionally, any items that caused translation difficulties, confusion, or were misunderstood in a specific language (either due to linguistic or cultural ambiguity) were revised. The revised items were once more translated into each language of the workgroup members and pilot tested. In cases where problems in item translatability or comprehensibility could not be resolved, the decision was made to permanently remove those items from the set. By adhering to this rigorous and iterative procedure, the consortium aimed to mitigate potential translation and culture-related issues that may have arisen when adapting an already developed instrument into different languages.

The proposed item sets were subsequently discussed with the central investigators, who checked content validity and absence of overlap with other (existing or recently developed) (sub)scales, thereby optimizing scale construction. Following that, suggested changes were communicated to the workgroup chairs who further deliberated upon these modifications within their respective workgroups. These changes were then implemented across all languages, followed by pilot testing. This iterative process was followed until all scales were developed.

Subsequently, language groups comprising native speakers of the respective languages undertook the task of translating and back translating all items that had not already been translated into the specific language within the workgroup responsible for their development. In this process, automated translation tools (e.g., DeepL [83]) were utilized, while caution was exercised to avoid complete reliance on automated translations. Additionally, the language groups evaluated the translatability of items of the pre-existing questionnaires that were integrated into the new generation of instruments, as well as of the new items. During language group meetings, issues concerning translations in specific languages were addressed by: (i) providing clearer indications of the intended meaning of the item to offer more translation alternatives without compromising its essence, (ii) modifying certain words in the item to enhance its translatability across various languages, or (iii) removing an item altogether if the preceding options proved ineffective. Subsequent alterations to new items were subject to review and discussion with the chairs of the respective workgroups responsible for their development. This iterative process continued until all translation-related issues were effectively resolved.

### Summary and research aims

In summary, ST has emerged as a prominent evidence-based treatment for personality and other complex syndrome disorders, steadily gaining widespread recognition. Nonetheless, the theoretical underpinnings of ST pose notable conceptual challenges, which restrict its universal application and the comprehensive assessment of schema-related constructs. Specifically, recent theoretical developments suggest that it would be fruitful to add specific fundamental emotional needs and their corresponding EMSs to Young’s original framework. Additionally, the differentiation between temperament dimensions and ways of coping in response to EMS activation appeared unsatisfactory in the existing SCI, necessitating a revision of the instrument. The extent to which the ST model of PD pathology applies universally across different cultures has also not been systematically examined, despite the assumption that the model possesses cross-cultural relevance. Lastly, while validated assessment tools for ST-related constructs have been developed and translated into multiple languages [e.g., 84–86], these instruments neither comprehensively encompass all crucial ST constructs nor do they fully capture the entire spectrum of PD pathology.

The present research project, conducted by an international consortium, aims to address these concerns. Specifically, the primary objectives are outlined as follows:

1] To develop revised instruments based on the reformulated theory concurrently in all countries and languages included in the project, enabling comprehensive testing of the reformulated theory across diverse cultures and linguistic backgrounds.
  - The Young Schema Questionnaire-4 (YSQ-4), assessing 23 hypothetically distinct EMSs.
  - The Schema Coping Inventory-Revised (SCI-R), assessing three ways of coping with schema activation (i.e., resignation, avoidance, and inversion).
  - The Schema Mode Inventory-3 (SMI-3), assessing 49 schema modes.
2] To assess the newly proposed taxonomy of EMSs, coping strategies, and SMs with particular focus on confirming the cross-cultural existence of EMSs, maladaptive ways of coping with EMS activation, and SMs as outlined in the reformulated model. This aim will be achieved by critically studying the psychometrics properties of the YSQ-4, SCI-R, and SMI-3 subscales, including (a) internal consistency; (b) unidimensionality; (c) cross-cultural invariance; (d) factorial validity (if possible) and (e) known-group validity.

The secondary objective is:

3) To examine the mediating role of coping with EMS activation in the relationships between EMSs and SMs (for a detailed description of mediation models, see [28]). It is expected that higher EMS scores will be associated with greater endorsement of maladaptive coping, which in turn will be positively linked to the frequency of SM occurrence. For instance, individuals who respond to the activation of the ‘Incoherent Identity’ schema through resignation coping are expected to show elevated scores on SMs such as ‘Confused Child’ and ‘Angry Child’.

The current study serves as the initial empirical phase of the project. Within this study, we will undertake the selection of optimal item sets per subscale, employing parallel analyses across all participating countries, with a minimum representation of 200 participants per country. The primary objective of this first international study is to establish internally consistent and unidimensional subscales of the three newly revised instruments (i.e., YSQ-4, SCI-R, and SMI-3) across all languages. The YSQ-4 and SMI-3 subscales should be relatively short, yet be reliable enough (i.e., Cronbach’s alpha ≥ .8), so that they can be used in clinical practice. Additionally, our goal is to ensure that intercorrelations among the subscales are as limited as possible based on the available empirical data. Lastly, the instruments’ ability to discriminate between clinical and non-clinical samples will be examined by means of known-group validity testing. In this regard, we expect participants from the clinical population to exhibit significantly higher scores on all (sub)scales compared to their non-clinical counterparts, except for the adaptive subscales (i.e., ‘Happy Child’ and ‘Healthy Adult’), where a reversed pattern is anticipated. Subsequent international studies, characterized by larger sample sizes, will further examine the validity of the resulting (sub)scales, which will be made available on an open-source platform for users across different countries.

## Materials and methods

### Setting

This international multi-site project is directed by researchers from the University of Amsterdam (UvA) and Maastricht University. A comprehensive list of all the participating sites, including universities and mental health care units, is provided as Supplementary Material in S1 Appendix to ensure transparency about the scope and diversity of data collection.

### Study design and ethical considerations

A non-experimental, cross-sectional design will be used with parallel sampling of non-patients and patients in more than 35 countries. An overview of the participating countries (and languages) is provided as Supplementary Material in S2 Appendix to document the breadth of cultural and linguistic representation in the data. This overview is preliminary, as other countries have also expressed interest in participating in this project and may be included in the future.

Approval to conduct this study has been obtained by the Ethics Review Board of the UvA, with the reference number: FMG-822_2022, in February 2023. The ethics committee letter of approval is provided as Supplementary Material in S1 File supporting the transparency and ethical integrity of our research. The UvA is the lead institution responsible for the administrative oversight of the overall project and the storage of the collected data from all participating countries. In addition, within each country, a designated researcher is assigned the responsibility of conducting data collection for the study within their respective country. This researcher is also responsible for obtaining local ethical approval from a duly recognized ethics review board operating within the country, if necessary, as most countries permit data collection through online systems provided that the study has received approval within the country where the data is stored.

Participation in the study will be entirely voluntary, and prospective participants will receive comprehensive information about the study’s objectives, procedures, privacy regulations, and their rights before providing written consent. They will also be informed of the option to complete the survey over multiple sessions within a 14-day time frame. Additionally, participants will be informed that, upon concluding the survey, they can download a report as compensation for their participation. This report will include their questionnaire scores, accompanied by brief descriptions of each scale, along with a disclaimer stating that a subset of the scores is based on (sub)scales that are still experimental and not yet psychometrically validated. Patients can share this report with their therapists if they consider it relevant. The information brochure will be available in all languages included in the project. The English version of the information brochure and consent form are provided as Supplementary Material in S2 File to ensure clarity on the informed consent process and adherence to ethical research standards.

Further, participants recruited from the general population (and clinical participants in certain developing nations) will be informed that, as compensation for their participation, they are also given the option to take part in a raffle to win a 50-euro voucher. This supplementary compensation is offered in acknowledgment of the possibility that non-clinical participants may not deem the report alone as a pertinent inducement for their involvement in the study. Those interested will be directed to a separate questionnaire in which they can provide a valid email address. This step is necessary to safeguard the anonymity of participants by separating their contact details from their responses to the main survey. After the raffle, all submitted email addresses will be permanently deleted. The English version of the information brochure and consent form used for respondents interested in participating in the raffle is provided as Supplementary Material in S3 File to document the consent process specific to raffle participation and support transparency in research practices.

### Status and timeline of the study

This project will be conducted in two phases. In the first phase the internal consistency, unidimensionality, cross-cultural invariance, as well as known-group validity of the three experimental versions of the measurement instruments will be tested. Malfunctioning items will be deleted, and items might also be deleted to shorten the scale whilst maintaining good enough psychometric properties. If the data acquired in this phase permits, we will also evaluate the factorial validity of these instruments. In the second phase (not described here), we will also assess the concurrent and convergent validity of the three scales. A separate study protocol will be submitted for the second phase of the project.

The data collection for the first empirical phase of the project is planned to commence in May 2024. The first version of the study protocol has already been submitted before the commencement of data collection. Data collection will continue until the recruitment goal is met, with an estimated duration of at least six months. Data analyses will be initiated once all the necessary data has been acquired. As part of their academic curriculum (e.g., supervised thesis project), university students may conduct preliminary analyses on subsets of the collected data.

### Power analysis

In the present study, we will recruit a minimum of 200 respondents per country which will lead to a total N of at least 6.000. This sample size will give ample opportunities for overall tests. The sample of 200 participants is sufficient for basic psychometric tests of unidimensionality and internal consistency, with around 8–12 items per subscale, per language and/or per country [87,88].

### Recruitment and study population

The recruitment of participants will be conducted by the research group in each participating country, as well as via a central website where a link to each language version of the survey will be provided. A range of convenience sampling methods will be used, including social media advertisements, flyers in community settings, personal invitations, student-led outreach, and snowball sampling. The recruitment process of mental health care patients will also be facilitated through established connections with collaborating mental health institutes and centres, including public and private clinics, hospitals, and outpatient services in both urban and rural areas. To promote diversity in sample composition, local research teams will be instructed to recruit participants varying in age, gender, socio-economic status, and cultural background, and will be encouraged to monitor demographic characteristics throughout the recruitment process to identify and address potential imbalances. Efforts will also be made to reach cultural minority groups, through relevant online platforms (e.g., expat Facebook groups) and clinics serving these populations where feasible. Finally, careful consideration will be given to ensure that the proportion of students recruited as part of the non-clinical sample does not exceed that of the clinical sample in each country.

To be eligible to participate in the study, individuals should meet the following criteria:

- Be 18 years of age or older at study entry.
- Have sufficient literacy in one of the languages involved in the project.
- Provide written informed consent.

Exclusion criteria include the presence of intellectual disability, functional illiteracy, or very limited cognitive skills that might hinder the ability to adequately comprehend the instructions and complete the survey accurately. Additionally, the status of being in the final stages of psychological treatment is deemed an exclusion criterion for known-group validity tests, as individuals nearing the conclusion of therapy and approaching recovery cannot be conclusively classified within either the clinical or non-clinical population. No other inclusion and/or exclusion criteria will be applied as the aim is to collect data from a broad sample of respondents.

### Assessment of sample characteristics

To obtain a comprehensive description of the participants and to further explore potential influences on test scores, basic demographic and mental health-related information will be collected. This will include data such as the participants’ age, cultural background, cognitive and intellectual status, functional literacy, treatment phase (if applicable), and mental health diagnostic status. The latter includes the presence and, when applicable, the broad classification of any mental health disorder diagnosis (e.g., anxiety disorders, mood disorders). In addition, to achieve balance in the representation of students across both non-clinical and clinical samples, a question will be included to determine the status of respondents as students. It should be noted that some of this information (i.e., cognitive and intellectual status, functional illiteracy, treatment phase) will be solely gathered to confirm the non-applicability of any exclusion criteria. In addition, the inquiry into participants’ cultural background not only serves demographic purposes but also involves the predefinition of majority and minority groups per country before conducting cross-cultural measurement invariance tests. A full item list is provided as Supplementary Material in S4 File, supporting full disclosure and enabling replication and/or adaptation in future research.

### Measures

#### Young Schema Questionnaire – 4.

The Young Schema Questionnaire – 4 (YSQ-4) is a self-report questionnaire, developed from the YSQ-3 [89]. This test version is comprised of 209 items aimed at evaluating the presence and severity of 23 EMSs. Each subscale is designed to assess a specific schema. The schemas included are emotional deprivation, abandonment/instability, mistrust/abuse, social isolation/alienation, defensiveness/shame, failure, dependence/incompetence, vulnerability to harm or illness, enmeshment/undeveloped self, entitlement/grandiosity, insufficient self‐control/self-discipline, subjugation, self‐sacrifice, approval-/recognition-seeking, unrelenting standards/hypercriticalness, emotional constriction, fear of losing control, negativity/pessimism, internal punitiveness, external punitiveness, unfairness, incoherent identity, and incomprehensible world.

A subset of items (N = 156) in the YSQ-4 is derived from the original YSQ-Long Form 3 [89]. To comply with guidelines promoting the use of non-discriminatory, gender-neutral language and enhance the translatability of the items across the languages included in this project, slight modifications were made to the wording of four original items of the YSQ-Long Form 3. For example, the original item from the ‘Defectiveness/Shame’ subscale, ‘*No man/woman I desire could love me once he/she saw my defects*’ was reformulated to ‘*No-one I desire could love me once my defects are revealed*’ to improve inclusivity.

In addition, 11 new items were developed by the central investigators and members of this project on the basis of the work by Yalcin et al. [69], according to which, Young’s original schemas of ‘Emotional Inhibition’ and ‘Punitiveness’ are both sub-divided into two discrete factors; the schema of ‘Emotional Inhibition’ is split into ‘Emotional Constriction’ and ‘Fear of Losing Control’, while the schema of ‘Punitiveness’ is divided into ‘Internal Punitiveness’ and ‘External Punitiveness’. A sample item from the ‘Fear of Losing Control’ subscale is: ‘*When I let my emotions go, things get out of hand*’, while an example from the ‘Emotional Constriction’ subscale is: ‘*It’s better to think than to feel*’. Similarly, a sample item from the ‘Internal Punitiveness’ subscale is: ‘*When something goes wrong, it is my fault*’ and from the ‘External Punitiveness’ subscale: ‘*Others should be punished for their mistakes and imperfections*’.

The YSQ-4 also contains 42 items devised by the project members to assess the schemas of ‘Unfairness’, ‘Incoherent Identity’, and ‘Incomprehensible World’ recently proposed by Arntz and colleagues [28]. Some example items include: ‘*I hold the belief that the world is an unfair place*’ (Unfairness), ‘*When I think about who I am, I feel chaotic*’ (Incoherent Identity), and ‘*I feel estranged from how society functions*’ (Incomprehensible World).

In this questionnaire, respondents are instructed to read each statement and indicate how accurately the statement currently describes them, using a 7-point scale ranging from 1 (‘*Completely untrue of me*’) to 7 (‘*Completely true of me*’). By adding up the scores for all items within each subscale and dividing by the total number of items in that subscale, a mean subscale score is obtained. Higher scores on each subscale reflect a higher endorsement of a particular EMS.

#### Schema Coping Inventory – Revised.

The Schema Coping Inventory-Revised (SCI-R) is the successor to the SCI [49], in which, with hindsight, conceptual problems were noted [28]. This experimental version consists of 36 items, organized into three subscales, each comprising 12 items. These subscales intend to assess the three different dysfunctional ways of coping with schema activation, namely resignation (formerly: surrender), avoidance, and inversion (formerly: overcompensation). Some example items are: ‘*I have thoughts about myself that easily overwhelm me*’ (resignation), ‘*I prefer to avoid facing my own emotional problems*’ (avoidance), and ‘*When a belief I dislike arises, I convince myself that the contrary is true*’ (inversion).

The SCI-R is a self-administered questionnaire in which respondents are asked to read each statement and indicate the extent to which they agree with it as a description of their general reactions. The ratings are anchored on a 7-point Likert scale ranging from 1 (‘*Completely disagree*’) to 7 (‘*Completely agree*’). A mean score on each subscale is obtained by adding the scores for all the items of the subscale and dividing this by the total number of items belonging to that scale. The higher the subscale score, the more a particular way of coping is endorsed.

#### Schema Mode Inventory – 3.

The Schema Mode Inventory-3 (SMI-3) encompasses self-descriptive statements designed to assess the frequency of schema mode manifestations. The test version consists of 20 subscales (210 items) derived from both the original SMI-1 [27] and the SMI-2 [25]. Several of these items were revised by the central investigators and members of this project to facilitate the translation of the items into the languages included in this project. For instance, the original ‘Enraged Child’ mode item on the SMI-2, ‘*When I’m angry, things can get so out of hand that people get hurt*’ was revised to ‘*When I’m angry, things can get so out of hand that people get physically hurt*’ to clarify that the intended meaning referred to physical rather than emotional harm—an important distinction that may not be explicit in all languages. Further, the instrument to-be-tested also contains 29 new subscales with 378 items. These were developed by the project members to assess the new schema modes proposed by Arntz and colleagues [28], as well as those emerging from systematically combining ways of coping with the new EMSs proposed by Yalcin and colleagues [69]. Some example items include: ‘*If I don’t win, it’s due to bad luck or an unfair system*’ (Winner), ‘*I confront other people with their shortcomings*’ (Punitive Lecturer), ‘*I do everything for others and forget about myself*’ (Subordinate Child), and ‘*I feel it’s inappropriate to be proud of myself*’ (Over-Humble). A list of the subscales intended to be constructed for the final version of the SMI-3 (i.e., operationalization of SMs) is provided as Supplementary Material in S3 Appendix and serves as a preregistration of the scale’s structure.

The items of the SMI-3 are scored on a 7-point Likert scale ranging from 1 (‘*Never*’) to 7 (‘*Always*’). A mean score is calculated from the subscale sum score divided by the number of items in that subscale. A higher score on each subscale indicates a more frequent occurrence of the corresponding mode.

### Data collection

Data will be collected in a computerized format via the online survey platform Qualtrics XM [90]. In regions where accessibility to web-based surveys is constrained, a paper-and-pencil rendition of the survey will be offered as an alternative. For each language, a separate version will be made. On the first page of the survey, potential participants will be presented with the information letter. Subsequently, they will be requested to provide informed consent and agree with the presented conditions to proceed further. The order of the questionnaires will be set so that participants will be first asked to provide demographic information before completing the experimental versions of the YSQ-4, SCI-R, and SMI-3 outlined above. To preclude possible order effects, the order in which the three ST-related questionnaires will be presented to the respondents will be randomized. Participants will also be able to go back and forth between questions. To ensure respondents’ engagement and accuracy of the collected data, the survey will include nine attention checks placed at intervals of every 60–100 items. Additionally, the ‘forced response’ option will be enabled in the web-based survey, requiring participants to respond to each question, thus reducing the likelihood of missing data. It is estimated that completing the survey on its entirety takes approximately two hours.

### Data management plan

To uphold participant privacy and maintain confidentiality, all data collected will be anonymized (i.e., there will be no link between research data and identifying data). Personal information collected only for short-term practical purposes (i.e., participants’ email addresses gathered solely for raffle participation) will be permanently deleted once the raffle concludes. The raw data and materials will be accessible only to members of the project team. The data will be shared with other researchers after the study is concluded or in data repositories. Publications will only report numerical data such as mean values and group statistics. Data will be obtained using the Qualtrics software company with which the UvA already holds an overarching privacy agreement. The data collected will be stored in cloud-based storage solutions that comply with the confidentiality, integrity and availability principles of the General Data Protection Regulation. The data management plan and privacy agreement have been reviewed and approved by the data steward and legal office of the UvA, respectively.

### Statistical analysis

In an initial data screening, range checks and descriptive statistics will be consulted to identify any potential inconsistencies, incomplete scale responses and/or data entry errors. Furthermore, an additional data screening process will be implemented to identify participants who meet one (or more) of the exclusion criteria.

Since the survey completed through the online software consists only of forced-response questions, we anticipate that the dataset will contain no item-level missing values. In addition, for the pencil-and-paper survey version, special attention will be directed towards ensuring that all questions are filled-out by the respondents. However, for both the computerized and the pencil-and-paper versions, missing data may still arise if participants discontinue the survey before completing one or more full scales. In such cases, the data will be retained for all scales up until the last successful attention check (see above). The remaining items will be coded as missing. Note that in the analyses described below, these missing data are relatively unproblematic as our analyses are conducted using pairwise deletion which retains most of the data even for participants without a full data record.

The quantitative variables will be summarized using descriptive statistics, including the mean, standard deviation, range, minimum, and maximum values. In instances where the data exhibits skewness, additional summary statistics including the median and quartiles will be reported. The association between numeric variables will be examined using either Pearson or Spearman correlation tests, depending on the distribution of the sample and the nature of the variables. To evaluate the internal consistency of the subscales and the higher-order scale, Cronbach’s alpha and McDonald’s omega will be calculated for each subscale. A preliminary analysis of the data will also be performed to ensure that all the assumptions required for confirmatory factor analysis are met. All aforementioned analyses will be conducted using R [91].

The main statistical analyses will be conducted using the lavaan package [92] within R [91]. Furthermore, for comparative group assessments, the multiple-group factor alignment method will be applied using Mplus [93,94]. In conducting the analyses outlined below, we will weight the cases by their representativeness for the corresponding country in terms of the variables age, gender, and education. We will use the iterative post-stratification algorithm as implemented in the ‘survey’ package in R [95].

Per subscale, confirmatory factor analysis (CFA) will be used to check the loadings of the individual items and the unidimensionality of the subscales. Several conventional goodness-of-fit indices will be employed to assess the model fit, including the root mean square error of approximation (RMSEA) [96], Tucker-Lewis Index (TLI) [97], and comparative fit index (CFI) [98]. Adequate fit criteria are determined as CFI and TLI values ≥ .90 and RMSEA values ≤ .08, following guidelines outlined in the literature [e.g., 99,100]. Where necessary, small model modifications (residual correlations among two items) will be considered, if these modifications are substantively interpretable (e.g., similar wording between two items).

Undesirable influences of language and country will be assessed by exploring measurement invariance [101,102]. Specifically, we will use the factor alignment method in Mplus [94,95] which is particularly suitable for testing measurement invariance across many groups as in the present study. In the alignment method the factor model parameters are estimated in all groups simultaneously in such a way that the amount of non-invariance is minimized. Using the estimates and corresponding standard errors, the factor loadings and thresholds are tested for significant differences (using a correction for multiple testing) and the items that are non-invariant are flagged. This is all automatized in Mplus. Items that demonstrate severe violations of invariance will be omitted from the corresponding scale unless there are theoretical reasons for their retention. As the alignment method assumes that the same factor structure holds in each group (configural invariance), we will check this assumption in a preliminary analysis using the model fit indices discussed above. In addition, it should be noted that the alignment method differs from the conventional approach which requires fitting a series of models that impose restrictions across groups [102]. With many groups, this approach becomes practically infeasible [94].

The internal consistency of each subscale will be assessed. When possible, we will remove items to reduce the length of the scales, all the while striving for maintaining a minimum internal consistency of .8.

Known-group validity will be assessed by comparing standardized factor means on the YSQ-4, SCI-R, and SMI-3 subscales between clinical and non-clinical samples. The hypothesized role of coping with schema activation (assessed with the SCI-R) in the relationship between schemas (assessed with the YSQ-4) and schema modes (assessed with the SMI-3) will be tested with a mediation analysis.

### Dissemination and analysis plan

Data analyses will commence upon the conclusion of data collection. We intend to disseminate the results of the study in the scientific community through publications in peer-reviewed scientific journals and presentations at scientific conferences. All active members of the consortium will be credited as co-authors in the study. Additionally, mental health professionals will be informed about the research findings through presentations at conferences that are attended by clinicians, both nationally and internationally. Furthermore, knowledge dissemination will also occur through the inclusion of chapters and books on ST and assessment procedures in ST. Lay summaries of the study’s findings will also be accessible to the public via the central website used for participant recruitment.

The ultimate objective is to psychometrically validate the updated versions of the three questionnaires related to ST in the participating countries using larger sample sizes. In this process, additional instruments will be incorporated to assess external validity. Subsequently, following this second phase, the revised instruments will be made accessible as open-source questionnaires through the websites of the International Society of Schema Therapy (ISST), as well as national ST associations. Following the completion of the second study phase, the pre-processed and fully anonymized data will also be made publicly available, subject to restrictions on non-commercial use and the requirement for appropriate citation.

## Discussion

Even though the ST framework was introduced over two decades ago, its theoretical underpinnings are far from being fully elucidated. Recent advancements in ST theorization have made significant contributions to our understanding of principal constructs (e.g., core emotional needs, EMSs) and have provided new impetus for comprehensive and empirically supported assessment instruments. Considering that the application of ST continues to expand across both Western and non-Western countries, there is a critical need for cross-culturally valid assessment instruments that encompass the entire spectrum of core ST constructs. Additionally, it is imperative that well-validated assessment tools are accessible and applicable across diverse countries to facilitate research endeavours. Hence, the overarching goal of this international project is twofold: first, to empirically examine the reformulated ST theory proposed by Arntz et al. [28], and second, to develop psychometrically robust ST instruments that are available in various languages and easily accessible to both researchers and clinicians worldwide, provided on an open-source basis.

In this paper, we describe a protocol concerning the first empirical study conducted to this end. This international study intends to determine optimal item sets for each subscale included in the revised versions of three widely used ST instruments (i.e., YSQ, SCI, and SMI) across different countries. Furthermore, the basic psychometric properties of each (sub)scale will be empirically tested. The findings of this study will serve as the basis for subsequent large-scale studies aimed at investigating the validity and factor structure of the new generation of ST-related instruments, as well as empirically examining models concerning the relationships between the three, core schema-related constructs.

### Strengths

The key strengths of this study lie in the academic and clinical profiles of the members involved in the international consortium conducting this research project and the approach employed to develop revised, extended versions of the three ST-related scales. The consortium includes experienced researchers and clinicians who have actively contributed to the empirical testing of the underlying theory of ST and possess ample experience in collaborating on large-scale international projects. In addition, several consortium members are esteemed experts in ST within their respective countries and/or possess expertise in psychometric evaluation of questionnaires, alongside having access to clinical sites crucial for recruiting individuals with mental health disorders. Finally, the consortium is complemented by a statistician (i.e., Molenaar) specialized in factor analysis with vast experience in lines of research directly pertinent to the current project.

Regarding the process of item development, a systematic, iterative, cross-cultural, and multi-language approach was used. This approach was chosen to eliminate potential risks of delay and address common translation issues often encountered in cross-cultural research, such as the lack of content equivalence in translated scales [103]. Another notable strength of this study pertains to the inclusion of a well-sized, mixed sample comprising a minimum of 200 participants per country, encompassing both individuals with and without a mental health disorder. This substantial sample size allows for comprehensive analyses and provides sufficient power for fundamental psychometric assessments, including tests of unidimensionality and internal consistency of the scales, as well as measurement invariance tests which will be conducted using the factor alignment method in Mplus to ensure that subscales are comparable across cultures.

Moreover, particular attention is paid to the recruitment of study participants, ensuring measures are in place to prevent homogeneous sampling (such as relying solely on the recruitment of university students) and instead aiming to obtain a culturally and socioeconomically diverse sample that is representative of the participating countries. A final strength of the present study is attributed to the approach adopted for data collection, which involves self-report measures administered through a computer-assisted, anonymous survey. This method is thought to promote the disclosure of sensitive and personal information (e.g., presence of mental health illness), while minimizing the risk of social desirability bias [104,105].

### Limitations

This study also has some limitations that should be noted. Firstly, self-selection bias is almost inevitable in studies that rely on voluntary participation. Although anonymity is guaranteed in our study, sociodemographic and other factors (e.g., severity of mental health conditions, presence of comorbidities), can potentially influence an individual’s decision to participate. Such biases could affect the generalizability of our findings, potentially leading to over- or under-representation of certain subgroups. To reduce this risk, a combination of recruitment strategies will be employed. In addition to online recruitment, personal invitations and snowball sampling will be used to increase outreach to individuals who may not otherwise engage with web-based research. Clinical participants will also be directly recruited through mental health facilities, enhancing the likelihood of including individuals with more severe or complex psychopathology who are typically underrepresented in voluntary studies. Moreover, participants will be offered the opportunity to enter a raffle for a small monetary reward, which may further incentivize participation across a broader range of sociodemographic backgrounds. In addition, to mitigate possible effects of such biases, we plan to collect sociodemographic and clinical information, which will allow for post-hoc statistical adjustments. Specifically, cases will be weighted to reflect the population distributions of age, gender, and education in each country.

Furthermore, while online participation confers manifold advantages, certain segments of the population or individuals lacking access to requisite technology or internet connectivity may be unable to partake in our study. To overcome this potential limitation, we have implemented several strategies to enhance the inclusivity and accessibility of the survey. First, we have provided a paper-and-pencil survey option in regions where web-based participation is impeded by technological or other constraints. In addition, we have endeavoured to optimize the online survey methodology by ensuring its compatibility with mobile devices, which are often more widely accessible among individuals with limited technological resources. Lastly, the enlistment of patients through mental health institutes and centres enables the provision of access to computers and technological assistance throughout the survey process. By employing these measures, we strive to address the digital divide and minimize the potential exclusion of certain population segments. However, we recognize that these solutions may not eliminate all barriers to participation.

Moreover, the presence of a mental health condition and its global classification (e.g., anxiety disorders, eating disorders, etc.) will be determined via self-reports provided by the participants themselves. Although self-reports may be subject to several self-serving biases that can affect the veracity and reliability of the provided information, this approach was chosen due to practical constraints and the convenience of data collection. To address this limitation, in future studies conducted as part of this project, we will implement other ways to document participants’ mental health status.

Another potential drawback of the present study pertains to the time commitment required for survey completion. Although attention checks have been implemented as a means of safeguarding the accuracy of the data gathered, and despite the flexibility afforded to participants in completing the survey over multiple sessions, there remains a possibility that the response burden and/or fatigue effects could contribute to inconsistent responses or jeopardize participants’ motivation to complete the survey in its entirety, potentially resulting in participant attrition.

### Challenges and mitigation strategies

Implementing this large-scale, cross-cultural study is anticipated to involve several logistical and methodological challenges. One primary concern relates to participant recruitment, particularly in light of the cultural diversity across sites. To enhance recruitment efforts, a multi-faceted dissemination strategy will be employed. Besides maintaining a central project website, the research team will organize webinars and podcasts to raise awareness and engagement. Outreach will also be conducted through international ST and practitioner networks, including professional groups on social media platforms. Supplementary video interviews will also be developed to further support recruitment. Moreover, to incentivize participation among recruiters, a clinical workshop on ST techniques will be offered to individuals who successfully recruit a minimum number of participants who complete the full assessment battery.

A further anticipated challenge relates to participant attrition, given the considerable length of the survey. To mitigate this, our protocol includes several strategies to support completion rates and ensure data quality. Attention checks will be embedded throughout the survey, and response data will be monitored in real time via the Qualtrics platform to identify valid submissions and assess recruitment progress. This will allow for timely adjustments to recruitment efforts to achieve target sample sizes in each participating country. Additionally, the standard 14-day completion window allotted to participants will be extended to up to 30 days when necessary, and the overall data collection period will remain flexible to accommodate variability in recruitment rates across sites.

### Implications

#### Scientific impact.

The scientific impact of this study involves innovating the theory underlying ST, thereby enhancing its comprehensiveness and consistency. This, in turn, will lead to a better coverage of personality-related pathology within the ST model and a deeper understanding of the relationships between its core constructs.

Additionally, conducting a systematic examination of the assumption regarding the cross-cultural universality of the ST framework is deemed crucial, particularly in light of previous studies that have either focused solely on linguistic adaptation and psychometric validation of individual ST instruments within specific cultural contexts or concentrated primarily on Western populations. While such studies offer preliminary support for the cross-cultural applicability of certain schema constructs, they often lack comprehensive theoretical integration or representative sampling across diverse populations. Consequently, questions remain about whether core ST constructs (e.g., schemas and modes) are conceptually equivalent and culturally resonant across different groups. This study contributes uniquely by systematically assessing the theoretical coherence and empirical consistency of ST constructs across diverse cultural contexts, addressing a critical yet underexplored aspect of schema theory’s universality and advancing the development of more universally valid therapeutic applications.

Moreover, there is an urgent need for a third-generation SMI, along with enhanced and expanded instruments to assess EMSs and coping strategies. This advancement is not only imperative but will also stimulate and propel future research endeavours. Lastly, our approach aligns with the current movement towards the development of transdiagnostic and dimensional models of (personality) psychopathology.

#### Clinical and societal impact.

Considering the high occurrence of personality pathology on a global scale and its profound ramifications for both individuals and society, a pressing need arises for the advancement of (cost-)effective interventions aimed at addressing PDs. The effectiveness and cost-efficiency of ST have been well-established in the treatment of diverse PDs. In the context of evidence-based ST protocols, the focal point lies in SMs. Therapists are bestowed with the task of identifying the specific SMs that underlie an individual’s symptomatology, subsequently employing targeted techniques aimed at addressing and alleviating these modes. The present project will fortify the empirical underpinnings and augment the cross-cultural validation of one of the most extensively utilized treatments for personality pathology. This endeavour will not only contribute to the dissemination of this treatment modality across different cultures but also enhance its accessibility to patients around the globe.

Additionally, many countries are witnessing a significant upswing in cultural diversity due to the growing population of immigrants and expatriates from various regions worldwide. While ST is offered to patients from diverse cultural backgrounds, it is implicitly assumed that the theoretical framework applies universally. This project seeks to critically evaluate and test the validity of this assumption in diverse cultural contexts.

As the project will also test important elements of the original and the reformulated theory, its findings will influence therapist training and practice. For instance, if the hypothesized schema of ‘Unfairness’ and its associated schema modes are confirmed, this will provide an empirical basis for training therapists to detect and treat clinical problems related to unfairness. In addition, already trained therapists will be offered information materials and advanced training to help them incorporate the new insights into their practice. Such advanced training could, for instance, focus on using Imagery Rescripting to address early experiences of unfairness. To reach this goal, the ISST board, and specifically the training coordinator, will be informed of the project’s findings, and discussions will take place regarding the implications for training programmes.

Finally, the availability of new open-access assessment instruments will provide valuable support to clinicians who encounter challenges in comprehending and treating individuals with complex PDs originating from different cultural backgrounds. To ensure continuous functionality, a dedicated website will be established through collaboration with the ISST. This website will serve as a platform for the maintenance and accessibility of these instruments, catering to the specific needs of mental health professionals.

## Conclusions

To conclude, personality pathology is a prevalent and complex phenomenon that imposes substantial impediments on individuals’ psychological functioning and presents a major therapeutic challenge for mental health professionals. In recent years, ST has gained global prominence as one of the most widely employed therapeutic approaches for individuals experiencing pervasive characterological mental health problems. Nonetheless, the cross-cultural implementation of ST across various PDs necessitates a critical re-evaluation of the theoretical basis of ST, the refinement of its related assessment instruments, and rigorous examination of its assumed universality.

Our planned study represents the initial stride towards the realization of multiple long-term objectives. That is, it aims to advance our understanding of the ST framework, broaden the application of ST to encompass a wider spectrum of PD pathology, foster research endeavours in ST and personality pathology, and facilitate the dissemination of evidence-based diagnosis and treatment of PDs.

Through our research, we aspire to make contributions towards identifying novel therapeutic targets and expanding the scope of ST to better address the diverse cultural backgrounds and clinical profiles of PD patients worldwide.

## Supporting information

S1 Appendix List of participating sites per country. (DOCX)

S2 Appendix Overview of participating countries and languages. (DOCX)

S3 Appendix Operationalization of schema modes. (DOCX)

S1 File Ethics committee approval letter. (PDF)

S2 File Information brochure. The English version of the information letter included in the first page of the (online) survey, along with the informed consent form.
(PDF)

S3 File Information brochure. The English version of the information letter intended for participants interested in joining the raffle, accompanied by the informed consent form.
(PDF)

S4 File Questionnaire sample characteristics. Questionnaire used for the assessment of respondents’ sociodemographic and mental health-related characteristics.
(PDF)
