# Supplementary material for: A next generation of the schema therapy model of personality pathology: A cross-cultural and international study protocol
Source: PLoS One. 2026 Jun 12;21(6):e0332723. doi: 10.1371/journal.pone.0332723 (PMC13262953; doi:10.1371/journal.pone.0332723)
Supplement: S1 Appendix — (DOCX) [file pone.0332723.s001.docx]

**S1 Appendix. List of participating sites per country.**

| **COUNTRY** | **SITE(S)** |
| --- | --- |
| Australia | - University of Western Australia, Crawley, Western Australia. - University of Technology Sydney, Sydney, New South Wales. - The University of Sydney, Sydney, New South Wales. - Mindwealth Psychology, Sydney, New South Wales. - Registered Schema Practitioners (various locations). |
| Bangladesh | - University of Dhaka, Dhaka. - CREA Psychiatric Clinic, Dhaka. |
| Belarus | - Minsk CBT Center, Minsk. - Belarusian State Pedagogical University named after Maxim Tank, Minsk. - Healthcare institution "Minsk City Clinical Center for Psychiatry and Psychotherapy", Minsk. - Regional state budgetary healthcare institution «Irkutsk City Hospital #6», Irkutsk, Russia. - Healthcare Institution “Brest city policlinic №2”, Brest. - Oncological dispensary of Baranovichi Healthcare institution «Baranovichi central polyclinic», Baranovichi. - Institute of Advanced Training and Retraining, Baranovichi State University, Baranovichi. |
| Belgium | - Psychologenpraktijk De Vest, Tienen. - Alexianen Zorggroep Tienen, Tienen. - Faculty of Psychology and Educational Sciences, KU Leuven, Leuven. - Child & Youth Institute, KU Leuven, Leuven. |
| Brazil | - Minas Gerais State University (UEMG), Minas Gerais. - Mindfluence Clinic, São Paulo, São Paulo. - Lilian Frossard Institute, Blumenau, Santa Catarina. |
| Bulgaria | - State Psychiatric Hospital "Sv. Iv. Rilski", Novi Iskar, Sofia. - Outpatient Mental health clinic "Adaptacia", Sofia. |
| China | - Northwest Normal University, Lanzhou, Gansu. - College of Science & Technology, Ningbo University, Ningbo, Zhejiang. - Anhui Sscience and Technology University, Chuzhou, Anhui. - Shanghai University of International Business and Economics, Shanghai. - New Life Psychiatric Rehabilitation Association, Hong Kong SAR. |
| Denmark | - Centre for Digital Psychiatry, Mental health services in the Region of Southern Denmark. - University of Southern Denmark, Odense. |
| Egypt | - Safe Haven Clinic, Alexandria. |
| France | - CEFTI Centre d’Etude et de Formation à la Thérapie Intégrative Bègles. - University of Bordeaux, Bordeaux. |
| Georgia | - Caucasus University, Tbilisi. - Tbilisi State University, Tbilisi. - Tbilisi State Medical University, Tbilisi. - Grigol Robakidze University, Tbilisi, Georgia. - M. Iashvili Children’s Central Hospital, Tbilisi. - Neurodevelopment Center, Tbilisi. |
| Germany | - LMU Munich, Munich. - Research Hospital, Max Planck Institute of Psychiatry, Munich. |
| Greece | - National and Kapodistrian University of Athens (NKUA), Medical School of Athens, 1st Psychiatric Clinic, Eginition Hospital, Specific Sector of Personality Disorders, Athens. - University of Macedonia, Thessaloniki. |
| Hungary | - Semmelweis University, Budapest. |
| India | - National Institute of Mental Health and NeuroSciences, Bangalore. - Center for Mental Health, Pune and Manoshanti Clinic, Pune. |
| Indonesia | - Gereja Kristus Di Indonesia. - HOPE worldwide Indonesia. - Atma Jaya Catholic University of Indonesia, Jakarta. - Atma Jaya Hospital, Jakarta. |
| Iran | - Siavoushan Psychological Center, Tehran. - Segal Psychological Services and Counseling Center, Tehran. - University of Science and Culture, Tehran. |
| Italy | - School of Cognitive Psychotherapy (SCP), Rome. - Association of Cognitive Psychology (APC), Rome. - University of Padua, Padova. |
| Latvia | - Riga Stradins University, Riga. |
| Lithuania | - Laboratory of Behavioral Medicine, Neuroscience Institute, Lithuanian University of Health Sciences, Kaunas. |
| Malaysia | - Hospital Kajang, Kajang, Selangor. - Amarantine Clinic, Wilayah Persekutuan, Kuala Lumpur. - International Psychology Centre, Kuala Lumpur. - International Psychology Centre & Complementary Medicine University, Kuala Lumpur. |
| Mexico | - Instituto Mexicano de Terapia de Esquemas, Mexico City. |
| Morocco | - Moroccan Institute of Cognitive Behavioural Therapy, Casablanca. - University Psychiatric Center, Marrakech. |
| Norway | - UiT The Arctic University of Norway, Tromsø. |
| Poland | - Community Mental Health Center, SWPS University, Poznań. - John Paul II Catholic University of Lublin, Lublin. - Centre for Psychiatry and Cognitive Behavioural Psychotherapy, Lublin. - Cognitive-Behavioural Therapy Clinic, SWPS University, Warsaw. |
| Portugal | - Psikontacto – Centre for Training and Therapeutic Intervention, Coimbra. - University of Coimbra, Coimbra. |
| Romania | - Babes-Bolyai University, Cluj-Napoca. - "Alexandru Ioan Cuza" University of Iași, Iași. - Becoming Center, Iași. - Socola Institute of Psychiatry, Iași. |
| Russia | - Moscow Institute of Schema Therapy, Moscow. |
| Singapore | - National University Hospital, Kent Ridge. |
| South Africa | - SAMRC Unit on Risk & Resilience in Mental Disorders, University of Cape Town. - Valkenberg Psychiatric Hospital, Cape Town. |
| South Korea | - Kyungnam University, Changwon. - Korean Schema Therapy Association, Changwon. - Y Psychological Counseling Center, Changwon. - Changwon Mental Health Center, Changwon. |
| Spain | - University of the Balearic Islands, Palma. - University of Deusto, Bilbao. - Son Espases University Hospital, Palma. - Institut d’Investigació Biomèdica de Girona-Institut d’Assitència Sanitària, Girona. |
| Switzerland | - Psychiatric University Clinic Zurich, Zurich. - University of Geneva, Geneva. - University of Zurich, Zurich. |
| Thailand | - Chiang Mai University, Chiang Mai. - Mahidol University, Nakhon Prathom. - Maejo University, Chiang Mai. - Suanprung Psychiatric Hospital, Chiang Mai. |
| The Netherlands | - University of Amsterdam, Amsterdam. - Maastricht University, Maastricht. - Tilburg University, Tilburg. - GGZ Oost Brabant, Helmond. - Behavioural Science Institute, Radboud University, Nijmegen. - Pro Persona Research, Nijmegen. - Vitaal GGZ, Nijkerk. - De Rooyse Wissel, Venray. |
| Türkiye | - Erenköy Mental and Nervous Diseases Training and Research Hospital (Psychotherapy & Addiction Center). - Bahçeşehir University, Istanbul. - Işık University, Istanbul. |
| UK | - King's College London, London. - GreenWood Mentors Ltd, Ryde. |
| USA | - Louis A. Faillace, MD, Department of Psychiatry and Behavioral Sciences, McGovern Medical School, The University of Texas Health Science Center at Houston (UTHealth Houston), Houston, Texas. - Schema Therapy Institute Midwest, Kalamazoo, Michigan. - Columbia University Vagelos College of Physicians and Surgeons, New York. - New York State Psychiatric Institute, New York. |
