# Supplementary material for: A next generation of the schema therapy model of personality pathology: A cross-cultural and international study protocol
Source: PLoS One. 2026 Jun 12;21(6):e0332723. doi: 10.1371/journal.pone.0332723 (PMC13262953; doi:10.1371/journal.pone.0332723)
Supplement: S2 Appendix — (DOCX) [file pone.0332723.s002.docx]

**S2 Appendix. Overview of participating countries and languages.**

| **COUNTRY (# 38)** | **LANGUAGE (# 32)** |
| --- | --- |
| Australia | English |
| Bangladesh | Bengali |
| Belarus | Russian |
| Belgium | Dutch |
| Brazil | Brazilian Portuguese |
| Bulgaria | Bulgarian |
| China | Simplified & Traditional Chinese |
| Denmark  Egypt | Danish  Generic Arabic |
| France | French |
| Georgia | Georgian |
| Germany | German |
| Greece | Greek |
| Hungary | Hungarian |
| India | Hindi |
| Indonesia | Bahasa Indonesia |
| Iran | Persian |
| Italy | Italian |
| Latvia | Latvian |
| Lithuania | Lithuanian |
| Malaysia | Malay |
| Mexico | Latin American Spanish |
| Morocco | Arabic |
| Norway | Norwegian |
| Poland | Polish |
| Portugal | European Portuguese |
| Romania | Romanian |
| Russia | Russian |
| Singapore | English |
| South Africa | English |
| South Korea | Korean |
| Spain | Spanish |
| Switzerland | German |
| Thailand | Thai |
| The Netherlands | Dutch |
| Türkiye | Turkish |
| UK | English |
| USA | English |
