# Supplementary material for: A next generation of the schema therapy model of personality pathology: A cross-cultural and international study protocol
Source: PLoS One. 2026 Jun 12;21(6):e0332723. doi: 10.1371/journal.pone.0332723 (PMC13262953; doi:10.1371/journal.pone.0332723)
Supplement: S3 Appendix — (DOCX) [file pone.0332723.s003.docx]

| **Schema Mode**  **S3 Appendix. Operationalization of schema modes.** | **Available Scale** | **Subscale intended to be constructed for the**  **SMI-3** |
| --- | --- | --- |
|  |  |  |
| ***Child modes*** |  |  |
|  |  |  |
| Abandoned Child | SMI-2 |  |
| Abused Child | SMI-2 |  |
| Lonely Child | SMI-2 | Disregarded Child |
| Disregarded Child | SMI-3 (Test version) |  |
|  |  |  |
|  |  | Non-Autonomous Child |
| Dependent Child | SMI-2 |  |
| Non-Autonomous Child | SMI-3 (Test version) |  |
|  |  |  |
|  |  |  |
| Intimidated Child | SMI-3 (Test version) |  |
| Parentified Child | SMI-3 (Test version) | Subordinate Child |
| Attention Demanding Child | SMI-3 (Test version) |  |
|  |  |  |
|  |  |  |
| Pessimistic Child | SMI-3 (Test version) |  |
| Inhibited Child | SMI-3 (Test version) | Constrained Child |
| Bad Child | SMI-3 (Test version) |  |
| Over-Diligent Child | SMI-3 (Test version) |  |
| Frightened/Panicking Child | SMI-3 (Test version) |  |
| Disappointing/Short-Falling Child | SMI-3 (Test version) |  |
|  |  |  |
|  |  |  |
| Victimized Child | SMI-3 (Test version) | Victimized Child |
|  |  |  |
| Confused Child | SMI-3 (Test version) | Confused Child |
| Disconnected child | SMI-3 (Test version) |  |
|  |  |  |
|  |  |  |
| Impulsive Child | SMI-1 | Impulsive Child |
| Spoiled Child | SMI-3 (Test version) | Spoiled Child |
| Grandiose Child | SMI-3 (Test version) | Grandiose Child |
| Undisciplined Child | SMI-1 | Undisciplined Child |
| ***Angry Child modes*** |  |  |
|  |  |  |
| Angry Child | SMI-1 |  |
| Aggrieved Child | SMI-3 (Test version) | Angry Child |
| Protesting Child | SMI-3 (Test version) |  |
| Enraged Child | SMI-1 | Enraged Child |
| Rebellious Child | SMI-3 (Test version) | Rebellious Child |
| Sulking Child | SMI-3 (Test version) | Sulking Child |
|  |  |  |
|  |  |  |
| ***Norm-setting modes (Parental modes)*** |  |  |
|  |  |  |
| Punitive Lecturer | SMI-3 (Test version) | Punitive Lecturer |
| Punitive Critic (Punitive Parent) | SMI-1 | Punitive Critic |
| Demanding Critic (Demandingg Parent) | SMI-1 | Demanding Critic |
|  |  |  |
|  |  |  |
| ***Avoidance Coping modes*** |  |  |
|  |  |  |
| Detached Protector | SMI-1 | Detached Protector |
| Funny Protector | SMI-3 (Test version) | Funny Protector |
| Angry Protector | SMI-3 (Test version) | Angry Protector |
| Avoidant Protector | SMI-2 | Avoidant Protector |
| Compliant Surrender | SMI-1 | Compliant Surrender |
| Reassurance Seeker | SMI-3 (Test version) | Reassurance Seeker |
| Detached Self-Soother | SMI-1 | Detached Self-Soother |
| Suspicious Over-Controller | SMI-1 | Suspicious Over-Controller |
|  |  |  |
|  |  |  |
| ***Inversion Coping modes*** |  |  |
|  |  |  |
| Hyper-Autonomous | SMI-3 (Test version) | Hyper-Autonomous |
| Perfectionistic Over-Controller | SMI-1 | Perfectionistic Over-Controller |
| Bully & Attack | SMI-1 | Bully & Attack |
| Attention & Approval-Seeker | SMI-2 | Attention & Approval-Seeker |
| Self-Aggrandizer | SMI-1 | Self-Aggrandizer |
| Clown | SMI-3 (Test version) | Clown |
| Pretender | SMI-3 (Test version) | Pretender |
| Conning & Manipulation | SMI-3 (Test version) | Conning & Manipulation |
| Idealizer | SMI-3 (Test version) | Idealizer |
| Daredevil | SMI-3 (Test version) | Daredevil |
| Slacker/Oblomov | SMI-3 (Test version) | Slacker/Oblomov |
| Pollyanna/Over-Optimist | SMI-3 (Test version) | Pollyanna/Over-Optimist |
| Over-Merciful | SMI-3 (Test version) | Over-Merciful |
| Overly Self-Permissive | SMI-3 (Test version) | Overly Self-Permissive |
| Over-Humble | SMI-3 (Test version) | Over-Humble |
| Emotional Excessiveness | SMI-3 (Test version) | Emotional Excessiveness |
| Emotional Daredevil | SMI-3 (Test version) | Emotional Daredevil |
| Predator | SMI-3 (Test version) | Predator |
| Winner | SMI-3 (Test version) | Winner |
|  |  |  |
|  |  |  |
| ***Functional/Adaptive modes*** |  |  |
|  |  |  |
| Happy Child | SMI-1 | Happy Child |
| Healthy Adult | SMI-1 | Healthy Adult |
