# Supplementary material for: A next generation of the schema therapy model of personality pathology: A cross-cultural and international study protocol
Source: PLoS One. 2026 Jun 12;21(6):e0332723. doi: 10.1371/journal.pone.0332723 (PMC13262953; doi:10.1371/journal.pone.0332723)
Supplement: S1 File — (PDF) [file pone.0332723.s004.pdf]

UNIVERSITY OF AMSTERDAM
Faculty of Social and Behavioural Sciences

Faculty Ethics Review Board

Faculty of Social and Behavioural Sciences
Nieuwe Achtergracht 166
1018 WV Amsterdam
The Netherlands
T + 31 (0) 20 525 58 20
www.fmg.uva.nl

**Date**	**Our reference**
02-02-2023	FMG-822
**Contact**	**Telephone**
Ethics Review Board	+31 (0) 20 525 58 [illegible]
**Subject**	
Project approval notice	

Dear Arnoud Arntz,

The research project titled "First psychometric evaluation of the revised YSQ, SMI, and SCI" complies with the guidelines formulated by the Ethics Review Board (FMG-UvA), University of Amsterdam, The Netherlands, and has been approved by the aforementioned Ethics Review Board on 02-02-2023.

Sincerely,

Arnold van Emmerik
Ethics Review Board
University of Amsterdam
