# Supplementary material for: A next generation of the schema therapy model of personality pathology: A cross-cultural and international study protocol
Source: PLoS One. 2026 Jun 12;21(6):e0332723. doi: 10.1371/journal.pone.0332723 (PMC13262953; doi:10.1371/journal.pone.0332723)
Supplement: S2 File — The English version of the information letter included in the first page of the (online) survey, along with the informed consent form. (PDF) [file pone.0332723.s005.pdf]

S2 File. Information brochure.

UNIVERSITEIT VAN AMSTERDAM

INFORMATION LETTER

Dear participant,

Thank you for considering participating in this study of the Faculty of Social and Behavioral Sciences of the University of Amsterdam! Before the study starts, it is important that you are informed about the procedures. Therefore, we would like you to read this information letter carefully. If anything might be unclear, feel free to contact the researcher through email: [Freideriki Carmen Mamali (f.c.mamali@uva.nl)]. The researcher will be happy to answer any questions you may have about this text or the general procedure.

Goal of the study

The aim of the present study is to evaluate new questionnaires that assess patterns in thoughts, emotions, and behaviours. These questionnaires are based on the theory that underlies schema therapy. Schema therapy is a relatively new psychological treatment for problems that are linked to childhood experiences and personality characteristics that developed because of these experiences. Both because of practical reasons and new scientific insights the existing questionnaires need to be updated. International experts from more than 30 countries collaborate in the project to develop new instruments that can be used in many languages and cultures. In the current phase we aim to test the questions that were developed for the new instruments. By doing this we can retain good questions and remove questions that do not do a good job.

In this study both people without psychological complaints and people with psychological complaints participate. For research reasons, it is essential that both groups participate.

Procedure

In this study, you will be asked to complete a set of questionnaires. The questions included pertain to the ways you view yourself, others, and the world. Other questions relate to the way in which you might deal with emotions and beliefs. Still other questions assess how often you experience specific ways of feeling, thinking, and behaving. Lastly, a few questions will be focused on demographic information (e.g., gender, age, level of education, and – if applicable – the kind of psychological complaints you are currently experiencing and the phase of treatment you might be presently receiving).

Throughout the study, you will have the flexibility to navigate between questions. The expected duration of participation ranges from 90 to 130 minutes. You have the option to complete the survey in one continuous session or break it into multiple sessions. If you opt for the latter, kindly remember that using the same link permits this, as long as you access the link from the **same electronic device and internet browser** within a span of **14 days**. To facilitate this option, a web cookie is automatically placed on your internet browser that keeps track of the survey progress. The web cookie is configured to expire 14 days from the moment you initiated the survey to safeguard anonymity.

Discomfort, risks, and insurance

This study is not expected to pose any foreseeable risks. However, in case you experience emotional discomfort or any negative consequences in response to answering the questionnaires, please contact a mental health professional or general practitioner within your region for advice.

Compensation

As compensation for your participation, you will receive a summary of your scores on all subscales of the questionnaires included in this study. You can download this summary at the end of the survey, after completing the questionnaires. If you are currently in treatment, you can give this summary to your practitioner/therapist, so that they can utilize it in your treatment.

In addition, as compensation for your participation, you can take part in a raffle to win a 50-euro voucher. After completing the questionnaires, you will be given the option to indicate if you would like to take part in this raffle. If you choose to do so, you'll be directed to another information letter about the raffle.

Voluntary participation

Your participation in this study is voluntary: you are not obligated to participate and there are no consequences if you decide not to participate in this study. During the study, you are also free to stop participating at any moment without any negative consequences (with the exception of not receiving a report summarizing your results) and without giving a reason. You can stop participating by exiting the online questionnaire administration. It's important to note that if you choose to stop or withdraw, we will not be able to delete your survey responses since they are anonymous and thus cannot be linked to you.

Your privacy is guaranteed

We do not collect any personal data in this study.

Data sharing

Your research/survey data will be analysed by the researchers that collected the information. The results of this study can be shared with other researchers and/or will be shared through a public database (open access), without any personal data. The results may be used in other future research, on a different topic than this study.

Retention period

Research data will be retained for at least 10 years.

Further information

Should you have questions about this study [FMG-822] at any given moment, please feel free to contact the responsible researcher; Freideriki Carmen Mamali (f.c.mamali@uva.nl). You can direct any formal complaints about this study to the Ethics Review Board (ERB) of the Faculty of Social and Behavioral Sciences of the University of Amsterdam using melding-ethiek-psy-fmg@uva.nl.

Thank you,

Freideriki Carmen Mamali, MSc.

Prof. dr. Arnoud Arntz

Prof. dr. Marleen Rijkeboer

Dr. Dylan Molenaar

University of Amsterdam

Universiteit van Amsterdam

CONSENT FORM

If you would like to participate in this survey, click on “Yes” below. With this you declare:

- I am 18 years or older.
- I have read and understood the information letter.
- I agree to participate in this study and I agree with the use of the data that are collected.
- I reserve the right to discontinue my participation from the study at any moment without providing any reason.
- I realize that the data I provide are anonymous, thus cannot be related to me as a person. Therefore, they cannot be deleted upon my request.

Yes, I agree to participate in the study
No, I do not agree to participate in the study
