# Supplementary material for: A next generation of the schema therapy model of personality pathology: A cross-cultural and international study protocol
Source: PLoS One. 2026 Jun 12;21(6):e0332723. doi: 10.1371/journal.pone.0332723 (PMC13262953; doi:10.1371/journal.pone.0332723)
Supplement: S3 File — The English version of the information letter intended for participants interested in joining the raffle, accompanied by the informed consent form. (PDF) [file pone.0332723.s006.pdf]

**S3 File. Information brochure.**

UNIVERSITEIT VAN AMSTERDAM

# INFORMATION LETTER

Dear participant,

Thank you for taking part in the FMG-822 study conducted by the Faculty of Social and Behavioral Sciences at the University of Amsterdam! You have expressed interest in entering a raffle for a chance to win a 50-euro voucher. Before proceeding, we kindly request you to carefully read the following information. Should you have any questions, please feel free to reach out to the researcher via email at [Freideriki Carmen Mamali (f.c.mamali@uva.nl)]. The researcher is available to address any questions you may have.

In order to participate in this raffle, you will only be asked to provide a valid email address. This is necessary because, upon the completion of the research project, one participant will be randomly selected as the winner of the 50-euro voucher. To contact the winner and send them the voucher, a valid email address is needed.

Your participation in the raffle is entirely voluntary. If you do not want to participate, choose the option ‘No, I do not agree to participate’ on the next page. There will be no negative consequences (except for not being included in the raffle) if you decide not to participate. You also do not have to provide a reason for not participating.

If you opt to withdraw your email address later, you can contact the researcher at f.c.mamali@uva.nl. Your email address will be then promptly deleted and no longer be utilized for the raffle. In that case, you withdraw your participation from the raffle and cannot win the voucher.

## Your privacy is guaranteed

We treat your personal data (i.e., email address), as required by law (the General Data Protection Regulation or GDPR). Once the raffle concludes and a winner is selected, the email address you provided will be permanently deleted.

Also note that the email address you provide here cannot be linked to the research data collected (i.e., your responses to the previous survey).

## Further information

Should you have questions about this study [FMG-822] at any given moment, please feel free to contact the responsible researcher; Freideriki Carmen Mamali (f.c.mamali@uva.nl). You can direct any formal complaints about this study to the Ethics Review Board (ERB) of the Faculty of Social and Behavioral Sciences of the University of Amsterdam using melding-ethiek-psy-fmg@uva.nl. If you have any complaints about the processing of your personal data, you can contact the Data Protection Officer of the University of Amsterdam through fg@uva.nl.

Thank you,

Freideriki Carmen Mamali, MSc.

UNIVERSITEIT VAN AMSTERDAM

Prof. dr. Arnoud Arntz

Prof. dr. Marleen Rijkeboer

Dr. Dylan Molenaar

University of Amsterdam

---

# CONSENT FORM

---

If you would like to participate in the raffle, click on “Yes” below. With this you declare:

- I have read and understood the information letter.
- I agree to participate in this raffle and I agree with the use of the data that are collected.
- I reserve the right to withdraw this consent at any moment without providing any reason.

**Yes, I agree to participate**
**No, I do not agree to participate**
