# Supplementary material for: A next generation of the schema therapy model of personality pathology: A cross-cultural and international study protocol
Source: PLoS One. 2026 Jun 12;21(6):e0332723. doi: 10.1371/journal.pone.0332723 (PMC13262953; doi:10.1371/journal.pone.0332723)
Supplement: S4 File — Questionnaire used for the assessment of respondents’ sociodemographic and mental health-related characteristics. (PDF) [file pone.0332723.s007.pdf]

S4 File. Questionnaire sample characteristics.

1. What is your age?

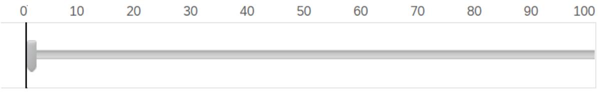

2. What is your gender?
   - Male
   - Female
   - Other (please specify)
   - I prefer not to say

3. Are you a student?
   - Yes
   - No

4. What is your highest level of completed education?
   - No education/early childhood education
   - Primary education
   - Lower secondary education
   - Upper secondary education
   - Post-secondary non-tertiary education
   - Short-cycle tertiary education
   - Bachelor’s or higher
   - Other (please specify)
   - I prefer not to say

5. What is your cultural background?
   - Dutch
   - ….
   - ….
   - ….
   - ….
   - ….
   - Mixed (two or more backgrounds)
   - Other (please specify)
   - I prefer not to say

6. Do you have any serious complaints concerning your mental health?
   - Yes
   - No
   - I prefer not to say

7. Could you please indicate which of the following applies to you? (You can choose more than one option.)
   - Personality Disorder (e.g., borderline personality disorder, avoidant personality disorder)
   - Bipolar or Related Disorder
   - Depressive Disorder
   - Anxiety Disorder (e.g., social anxiety disorder, generalized anxiety disorder)
   - Trauma- or Stressor-Related Disorder (e.g., PTSD, acute stress disorder)

- Dissociative Disorder (e.g., depersonalization/derealization disorder, dissociative identity disorder)
- Neurodevelopmental Disorder (e.g., autism spectrum disorder, attention-deficit/hyperactivity disorder)
- Schizophrenia Spectrum or Other Psychotic Disorder (e.g., schizophrenia, delusional disorder)
- Obsessive-Compulsive or Related Disorder (body dysmorphic disorder, hoarding)
- Somatic Symptom or Related Disorder (e.g., illness-anxiety disorder, conversion disorder)
- Feeding or Eating Disorder (e.g., anorexia nervosa, bulimia nervosa)
- Substance-Related or Addictive Disorder (e.g., alcohol use disorder, cannabis use disorder)
- Other (please specify)
- I prefer not to say

8. If you are undergoing treatment for a mental health complaint, which phase of treatment do you feel you are currently in?
    - Waiting to receive treatment
    - Early phase/beginning of treatment
    - Middle phase of treatment
    - Final phase/nearing treatment completion
    - Currently **not** undergoing any treatment for any complaint concerning my mental health
    - I prefer not to say

9. Do you have an intellectual disability or difficulties with reading and understanding written text?
    - Yes
    - No
    - I prefer not to say
